# Obesity matters but is not perceived: A cross-sectional study on cardiovascular disease risk factors among a population-based probability sample in rural Zambia

**DOI:** 10.1371/journal.pone.0208176

**Published:** 2018-11-29

**Authors:** Yukiko Tateyama, Teeranee Techasrivichien, Patou Masika Musumari, S. Pilar Suguimoto, Richard Zulu, Mubiana Macwan’gi, Christopher Dube, Masako Ono-Kihara, Masahiro Kihara

**Affiliations:** 1 Department of Global Health and Socio-epidemiology, School of Public Health, Graduate School of Medicine, Kyoto University, Kyoto, Japan; 2 Medical Education Center, Graduate School of Medicine, Kyoto University, Kyoto, Japan; 3 Institute of Economic and Social Research, University of Zambia, Lusaka, Zambia; 4 Ndola District Health Office, Ndola, Zambia; University of Zurich, SWITZERLAND

## Abstract

**Background:**

Sub-Saharan Africa, including Zambia, has experienced an increase in overweight and obesity due to rapid lifestyle changes associated with recent economic growth. We explored the prevalence and correlates of overweight and obesity in rural Zambia. We also investigated the role of self-perception of body weight in weight control given the local socio-cultural context.

**Methods:**

In this cross-sectional study, we recruited 690 residents of the Mumbwa district aged 25–64 years through a multistage, clustered, household random sampling. We administered a questionnaire and collected anthropometric and bio-behavioral data from May to July 2016. Factors associated with body mass index (BMI) ≥25 kg/m^2^ and underestimation of body weight were assessed using multiple logistic regression.

**Results:**

Of the weighted sample of 689 participants (335 men and 354 women), 185 (26.8%) had BMI ≥25 kg/m^2^. In multivariate analyses, female gender, age 45–64 years, tertiary education, higher fruit and vegetable intake, high blood pressure, abnormal blood lipid profile, and Hemoglobin A1c ≥5.7% were significantly associated with BMI ≥25 kg/m^2^. Among participants with BMI ≥25 kg/m^2^, 14.2% and 58.2% perceived themselves as being underweight and normal weight, respectively. Age 45–64 years was the only factor significantly associated with body weight underestimation. Preference for obesity was reported by 17.5% and 3.6% of respondents with BMI <25 kg/m^2^ and BMI≥25 kg/m^2^, respectively; “looks attractive” and “fear of being perceived as HIV-positive” were the main reasons.

**Conclusion:**

In rural Zambia, overweight and obesity are prevalent and significantly associated with alterations in blood pressure, blood lipid profile, and glucose metabolism. However, most subjects with BMI ≥25 kg/m^2^ underestimated their body weight; some preferred obesity, in part due to cultural factors and HIV-related stigma. A health promotion program that addresses such perceptions and body weight underestimation should be urgently introduced in Zambia.

## Introduction

The epidemic of overweight and obesity is an important global health concern. The prevalence of overweight and obesity among adults has doubled since 1980 worldwide [1], and it has increased in many low- and middle-income countries (LMICs). Cardiovascular diseases (CVDs), diabetes, musculoskeletal disorders, and some types of cancers that are associated with overweight and obesity are the leading causes of mortality worldwide [2]. Accordingly, the increase in overweight and obesity can pose a huge burden on healthcare systems. In fact, it has been projected that the burden of chronic diseases will become comparable to the burden of acute infectious diseases in the near future in many LMICs [3,4].

Sub-Saharan Africa (SSA), including Zambia, has also been experiencing an accelerating rate of overweight and obesity [5–7]. Obesity-related diseases currently represent 3.8% of disability-adjusted life years (DALYs) [8]. Although Zambia is still greatly affected by epidemics of infectious diseases, especially HIV, with a prevalence of 13.1% in 2013–2014 [9], the estimated national prevalence of overweight and obesity in Zambia was 29.2% in 2014, compared with 26.4% in 2010 [10].

The rapid increase of overweight and obesity in Africa has been ascribed to changes in diet and lifestyle brought on by economic growth, rapid urbanization, and globalization [5,11–13]. In the SSA context, perceptions surrounding body weight should be explored as a moderator. In many SSA societies, obesity is culturally accepted or even desirable, particularly for women [14–16]. However, research on overweight and obesity remains scarce both in amount and depth in many SSA countries, including Zambia [5]. In Zambia, prevalence studies have been conducted only in a few districts including the capital city, with the results already outdated in view of rapid social changes [6,17,18]. There have been no studies that involved biological measurements to assess the metabolic effects of overweight and obesity or have evaluated the perception of overweight and obesity in consideration of the local cultural context in Zambia.

Thus, we designed a sequential mixed methods study. The initial qualitative phase was conducted in August–September 2014 with 67 rural residents of Zambia participating to explore their lifestyle, particularly dietary habits and perception about body weight. This phase revealed a set of factors related to overweight and obesity such as excessive sugar, salt, and cooking oil intake; cultural preference for obesity; and body image stigmas related to HIV (unpublished data).

Based on our prior qualitative study, the current study was designed as the second quantitative phase of the mixed methods study to confirm our major findings in the same community. Using a probability sample, this study aimed to estimate the prevalence of overweight and obesity among residents and analyze the socio-cultural and bio-behavioral correlates of overweight and obesity as well as perceptions about overweight and obesity, in order to inform future CVD prevention programs in Zambia.

This study found that overweight and obesity were prevalent among the targeted rural community of Zambia, were significantly associated with cardio-metabolic disorders but were largely underestimated by the participants.

## Methods

### Study setting and participants

This study was conducted in May–July 2016. Zambia is a land-locked country in southern Africa. The Mumbwa district in the central province was selected as our study area because it is a typical rural area experiencing urbanization and economic growth but has maintained traditional culture. The district is located 150 km west of the capital, Lusaka. It is home to approximately 210,847 inhabitants; 15% live in semi-urban areas and 85% are in rural areas [19].

The target population included male and female residents aged 25–64 years. Since the objective of this study was to investigate lifestyle-related risk factors, only residents who had been living in the study area for ≥6 months and had adopted the lifestyle of the study area were included. Pregnant women and women who had given birth in the last 6 months were excluded because of potentially different dietary habits and lifestyles and the fact that prepartum and postpartum weight could affect anthropometric and biological data.

### Study design and sampling

**Fig 1:**
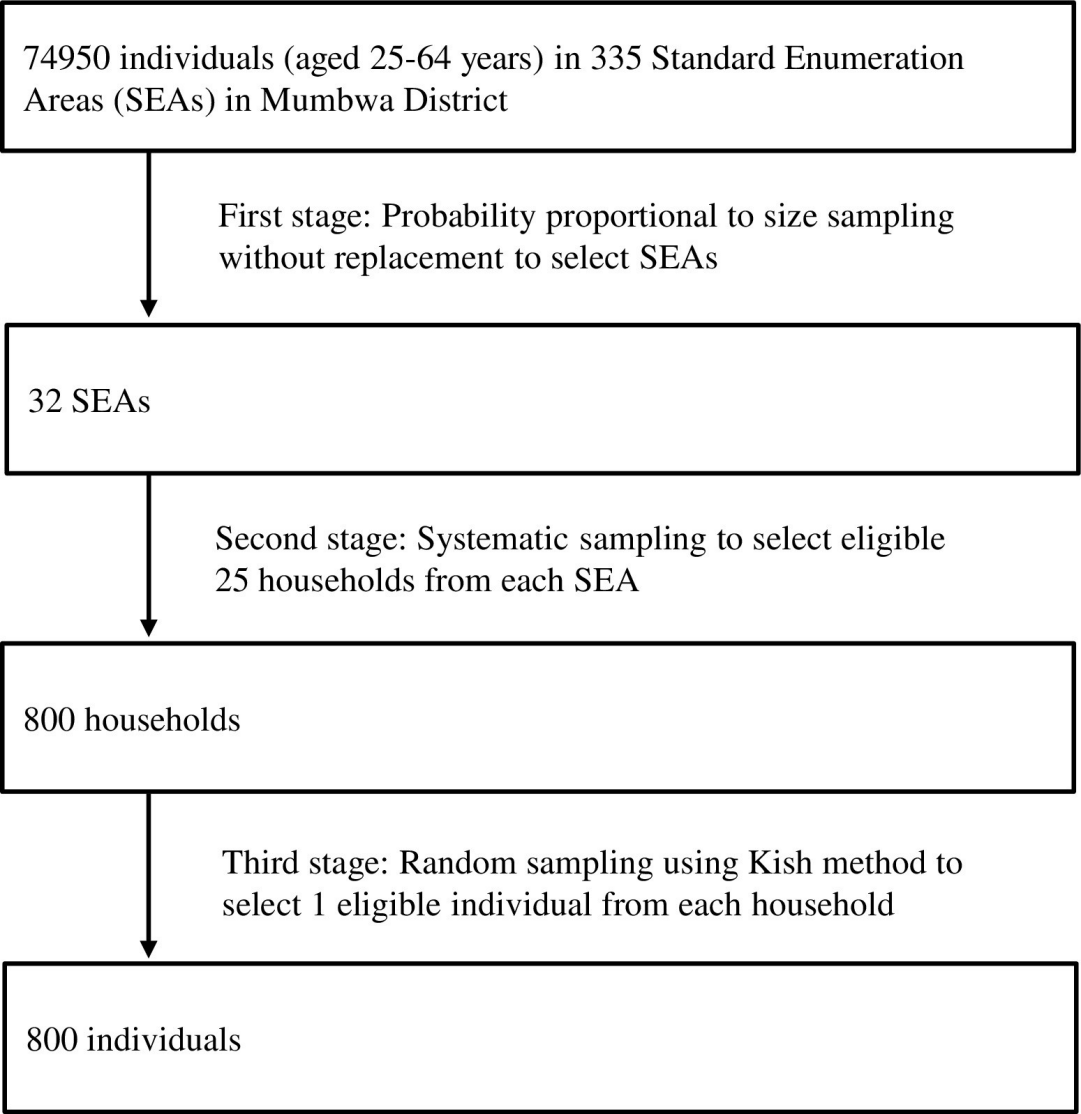
Sampling procedure according to probability proportional to size method.

This cross-sectional study employed a three-stage probability proportional to size (PPS) cluster sampling as shown in Fig 1. The sample size was calculated based on the recommendations of the WHO STEPwise approach to surveillance (STEPS) [20], assuming 95% confidence level, 5% margin of error (e^2^) and 30% prevalence of hypertension in rural areas [21]. The minimum sample size required was 167 subjects, which was increased to 800 to address design effects (loss of sampling efficiency due to cluster sampling), an assumed 20% non-response rate, and planned subgroup and multivariate analyses.

A list of study sampling clusters and Standard Enumeration Areas (SEAs) was provided by the Central Statistical Office (CSO) of Zambia. In the first stage, 32 SEAs were selected through PPS sampling without replacement using the latest sampling frame of the Zambia Population and Housing Census 2010 [19]. In the second stage, within each selected SEA, field staff, consisting of mappers from CSO and research assistants, conducted mapping and made a list of all households. Then, field staff visited all listed households to explain the survey and research objectives to seek permission to list all eligible members in each household. A total of 25 households were selected through systematic sampling in each SEA. In the third stage, within each selected household, only one individual was selected using the Kish Household Coversheet based on the WHO STEPS [20]. We scheduled a date and place to administer the questionnaire survey and make anthropometric and biological measurements in each SEA with consideration of the participants’ convenience. Taking into account the need for fasting starting at 8 pm on the day before biological measurements, we met with all the recruited individuals (or their family members or closest neighbors, if absent) 1–2 days before testing to inform them about fasting and coming to the testing venue on the scheduled date.

### Questionnaire

We developed the questionnaire (S1 Appendix) based on previous Zambian and international studies [22–24], as well as the results of our prior qualitative study in 2014 that revealed marked knowledge and practice gaps in lifestyle-related risk factors for CVDs and unique perceptions of body weight affected by cultural traditions and the HIV epidemic (unpublished data) as indicated in the corresponding question areas described below. The items of English questionnaire were translated into three local languages (Bemba, Nyanja, Tonga) to standardize the interview in local languages (S2 Appendix). It was then pilot-tested in March 2016 among 60 Mumbwa or Kabwe residents to ensure face validity and assess test-retest reliability with one week interval. Participants in the pilot testing were not included in the main study. Kappa coefficients were calculated for categorical variables and intra-class correlation coefficients were calculated for continuous valuables. All variables used in the analysis demonstrated acceptable reliability of ≥0.50 (*p* value<0.05).

The questionnaire included items on:

1. Socio-demographic variables, such as age, marital status, educational level, monthly income (10 items).
2. Food insecurity, which was assessed using the Household Food Insecurity Access Scale (HFIAS) [25]. The HFIAS is a validated instrument that has been shown to discriminate between food security and insecurity across different cultural contexts. The scale consists of nine "occurrence" and "frequency" questions. Food security status was presented in the following categories: food secure, mildly food insecure, moderately food insecure, and severely food insecure, which we dichotomized into "severely" or "not severely" food insecure. Cronbach’s alpha was 0.89, demonstrating that the scale had high internal consistency in our sample.
3. Psychological distress, which was assessed using the Kessler-6 scale, a standardized and validated screening tool for non-specific psychological distress (depression and anxiety disorders) [26]. The scale is comprised of six items administered on a five-point Likert scale ranging from 0 (none of the time) to 4 (all of the time). Participants were asked to rate how frequently they felt nervous, hopeless, restless or fidgety, depressed and nothing could cheer them up, everything was an effort, and worthless. Scores ≥13 indicate a higher probability of psychological distress. Cronbach’s alpha was 0.65, suggesting that the scale had moderate internal consistency in our sample.
4. Perceptions and preferences about overweight and obesity, which were assessed using questions based on the results of our prior qualitative study. The question about perception was used to identify how participants perceived their body weight from the following options: underweight, normal, overweight, or very overweight (obese). The questions about preference asked whether participants preferred being overweight or not and reasons, if applicable (9 items).
5. Dietary habits, which were assessed with questions about intake of fruits and vegetables, salad oil and fat, salt, and sugar based on the results of our prior qualitative study. For fruits and vegetables, the frequency of consumption per week on average was asked. For salad oil and fat, salt, and sugar, the amount (bottle or weight) consumed per day was ascertained (22 items).
6. Physical activity, which was assessed with questions about the number of days per week that involved work, sports, walking, or bicycling for at least 10 minutes continuously (13 items).
7. Other variables included self-reported medical history and current medications (4 items) and the frequency of tobacco and alcohol use (9 items).

### Anthropometric and biological measurements

Height and weight were measured with the subjects not wearing thick clothes or shoes. Weight was measured to the nearest 100 grams using an electronic scale (OMRON HBF-223-G, OMRON Corporation, Kyoto, Japan). Height was measured to the nearest 0.1 cm using a tape measure. Body mass index (BMI) was calculated using weight in kilograms and height in meters squared, and classified into four categories following WHO guidelines [2]: underweight (<18.5 kg/m^2^), normal (18.5–24.9 kg/m^2^), overweight (25–29.9 kg/m^2^), and obese (≥30 kg/m^2^). Hip and waist circumference were measured using a tape measure. The hip-to-waist ratio was classified into the categories of <0.85, 0.85–0.899, and ≥0.90 [20,27].

Blood pressure was measured using an automated digital blood pressure monitor (OMRON HEM-7130-HP, OMRON Corporation). Three blood pressure measurements were taken at 3-minute intervals in a seated position after 15 minutes of rest. The average of the last two readings was used. High blood pressure was defined as either systolic blood pressure (SBP) ≥140 mmHg or diastolic blood pressure (DBP) ≥90 mmHg [10].

Hemoglobin A1c (HbA1c), total cholesterol, low-density lipoprotein cholesterol (LDL-cholesterol), high-density lipoprotein cholesterol (HDL-cholesterol), and triglycerides were measured using finger prick blood samples obtained using medical finger-stick devices (Cobas b 101, Roche Diagnostics K.K., Tokyo, Japan). HbA1c was categorized as normal (<5.7%), prediabetes (5.7–6.4%), and diabetes (≥6.5%). An abnormal blood lipid profile was defined as at least one of the following: total cholesterol ≥220 mg/dl, LDL-cholesterol ≥140 mg/dl, HDL-cholesterol ≤40 mg/dl, or triglycerides ≥150 mg/dl.

Spot urine samples were collected from all participants. Urine glucose was measured using a test strip. The sodium to potassium ratio (Na/K) was assessed using a handheld urinary-Na/K ratio monitor (HEU-001F, OMRON Corporation).

### Data collection

To ensure high-quality data collection, we recruited field staff who had tertiary education and prior field survey experience. Field staff attended the practice session during the pilot testing phase and a two-day intensive training session to understand the study objectives, methods, and interview procedures. Face-to-face interviews were carried out by field staff at a venue preferred by the participants, such as the participant’s home, community meeting place, or school. In addition, licensed nurses were recruited and trained to collect anthropometric measurements (height and weight, blood pressure, hip and waist circumference) and biological samples (blood and urine samples).

### Statistical analysis

The data were analyzed using the Complex Sample module in IBM SPSS Statistics version 21 (IBM Corp., Armonk, NY, USA) to adjust for the effects of multistage sampling, clustering, and weighting. Sample weights accounted for different selection probabilities at each sampling stage, the non-response rate in each SEA, and post-stratification adjustments to correct for differences between our sample and the district population estimates based on the 2010 census. Total weights were standardized as the final weight.

Bivariate analyses were performed to investigate associations between independent variables (socio-demographic and lifestyle-related variables, food security, and biological and anthropometric variables) and BMI ≥25 kg/m^2^ (overweight and obesity) using a variant of the second-order Rao-Scott adjusted chi-square statistic. Variables that were associated with BMI ≥25 kg/m^2^ at *p*<0.10 and variables that are epidemiologically important to overweight and obesity were included in multiple logistic regression models. Marital status was also excluded due to the low number of participants that were single in each cell when stratified by BMI category. In addition, bivariate and multiple logistic regression analysis were performed to evaluate the associations between demographic variables and body weight underestimation among participants with BMI ≥25 kg/m^2^. Although 29.9% of participants did not fast despite advance instructions to do so, they were included in the analysis because there were no statistically significant differences in any of the biological measurements between participants who fasted versus did not fast based on the *t*-test (all *p*>0.10, except for total cholesterol, which was slightly (12 mg/dl) but significantly lower (*p*<0.01) in participants who did not fast).

### Ethical considerations

This study was approved by the Ethics Committee of the Graduate School and Faculty of Medicine of Kyoto University, Japan (R0403) and ERES Converge, Zambia (No. 2016-Jan-003) for the pilot phase. The University of Zambia Biomedical Research Ethics Committee, Zambia (No. 011-02-16) and the National Health Research Authority, Zambia (MH/101/23/10-1) granted approval for the main survey. All participants gave written informed consent prior to study participation.

## Results

Of 800 participants recruited, 712 participants agreed to participate (89% response rate). The most commonly reported reasons for non-response were lack of interest in the study and time conflicts with work. We excluded 22 participants from the analyses due to either missing interview, anthropometric, or biological data. There were no significant differences in gender and age distribution between all participants (n = 712) and those included in the analysis (n = 690). One hundred eighty-five participants (26.8%) were overweight or obese.

The demographic characteristics of the study population are shown in Table 1. The final sample included 335 (48.6%) men and 354 (51.4%) women. Mean age was 41.9 years (SE, 0.55 years) and 429 (62%) were aged 25–44 years. Most participants were married (80.8%), had only up to primary education (74.3%), and were self-employed (69.7%). Approximately half of the participants (47.4%) had a monthly income of 50 USD or less, and 27.7% experienced severe food insecurity. Of all participants, 8.0% were diagnosed with hypertension (self-reported), and 10.4% were living with HIV (self-reported). The proportion of female participants with BMI ≥25 kg/m^2^ was 36.7%, compared with 16.1% among male participants.

**Table 1:** Demographic characteristics of study participants in a rural district of Zambia.

	Male			Female			Total		
	n	% (95% CI)		n	% (95% CI)		n	% (95% CI)	
**Number**									
Unweighted	332			358			690		
Weighted	335			354			689		
**Socio-demographic data**									
Age, years (SE) Age group (year)	42.7	(0.76)		41.1	(0.73)		41.9	(0.55)	
25–44	203	60.6	(54.9,66.0)	226	63.9	(56.7,70.5)	429	62.3	(57.4,66.9)
45–64	132	39.4	(34.0,45.1)	128	36.1	(29.5,43.3)	260	37.7	(33.1,42.6)
Marital status									
Single	20	5.8	(3.1,10.6)	7	2.0	(1.0,4.1)	27	3.9	(2.3,6.4)
Married	300	89.4	(84.0,93.2)	257	72.6	(67.1,77.4)	557	80.8	(76.6,84.3)
Divorced/widowed	16	4.8	(2.9,7.8)	90	25.4	(20.9,30.4)	106	15.4	(12.6,18.6)
Education level									
≤Primary	229	68.3	(59.2,76.1)	284	80.1	(69.8,87.5)	513	74.3	(65.8,81.4)
Secondary	77	23.0	(17.9,29.1)	50	14.2	(9.4,20.9)	127	18.5	(14.3,23.5)
≥Tertiary	29	8.7	(3.8,18.8)	20	5.7	(2.5,12.3)	49	7.2	(3.4,14.5)
Employment status									
Employed	58	17.2	(11.1,25.7)	28	7.8	(4.9,12.2)	85	12.4	(8.2,18.2)
Self-employed	255	76.2	(67.2,83.4)	225	63.6	(55.1,71.3)	481	69.7	(62.3,76.3)
Unemployed/retired	22	6.6	(4.1,10.4)	101	28.6	(22.1,36.1)	123	17.9	(14.1,22.4)
Monthly income (US Dollar)^a^									
≤50	157	46.9	(38.3,55.8)	169	47.8	(39.9,55.7)	326	47.4	(40.6,54.2)
>50	178	53.1	(44.2,61.7)	185	52.2	(44.3,60.1)	363	52.6	(45.8,59.4)
Food security									
Secure	107	32.1	(26.9,37.7)	85	23.9	(19.1,29.6)	192	27.9	(23.7,32.5)
Mildly insecure	25	7.4	(5.2,10.4)	21	5.8	(3.4,9.8)	45	6.6	(4.7,9.2)
Moderately insecure	132	39.4	(33.7,45.4)	129	36.4	(29.5,44.0)	261	37.9	(32.8,43.3)
Severely insecure	71	21.2	(16.8,26.3)	120	33.8	(26.8,41.6)	191	27.7	(22.8,33.1)
**Medical history (self-reported)**									
Hypertension	18	5.4	(2.7,10.5)	37	10.4	(6.9,15.5)	55	8.0	(5.6,11.2)
Diabetes	3	0.9	(0.3,2.9)	2	0.6	(0.1,2.3)	5	0.7	(0.3,1.8)
HIV infection	28	8.4	(5.6,12.6)	43	12.2	(8.9,16.5)	71	10.4	(7.9,13.5)
**Weight status**									
Body mass index (kg/m^2^)									
<18.5	28	8.4	(5.4,13.0)	25	7.1	(4.6,10.9)	53	7.8	(5.9,10.2)
18.5–24.9	253	75.5	(69.0,80.9)	199	56.2	(49.5,62.6)	452	65.5	(60.6,70.1)
25–29.9	40	12.0	(7.7,18.0)	80	22.6	(18.0,28.1)	120	17.4	(13.9,21.6)
≥30	14	4.1	(2.3,7.2)	50	14.1	(10.2,19.2)	64	9.2	(7.1,12.0)

Percentages might not sum to 100 due to rounding.

Some discrepancies in the numbers are also due to rounding.

CI: confidence interval

^a^ Dichotomized at the minimum wage

Variables significantly associated with BMI ≥25 kg/m^2^ in the bivariate analysis included gender, age, alcohol use, smoking, involvement in physical activities, psychological distress, blood pressure, waist-to-hip ratio, HbA1c, blood lipid profile, and self-reported hypertension and diabetes (Table 2).

**Table 2:** Bivariate correlates of BMI ≥25 kg/m^2^ in a rural district of Zambia.

	BMI <25 kg/m^2^			BMI ≥25 kg/m^2^			
	(n = 505)			(n = 185)			
	n	% (95% CI)		n	% (95% CI)		*p* value
**Socio-demographic characteristics**							
Gender							
Male	281	55.6	(49.5,61.5)	55	29.5	(22.2,38.0)	<0.01
Female	224	44.4	(38.5,50.5)	130	70.5	(62.0,77.8)	
Age group (year)							
25–44	339	67.2	(62.6,71.5)	90	48.7	(38.2,59.4)	<0.01
45–64	166	32.8	(28.5,37.4)	95	51.3	(40.6,61.8)	
Marital status							
Single	24	4.7	(2.6,6.0)	3	1.7	(0.6,4.7)	0.05
Married	411	81.5	(76.6,85.5)	145	78.8	(71.9,84.4)	
Divorced/widowed	70	13.8	(10.9,17.5)	36	19.5	(14.0,26.5)	
Education level							
≤Primary	387	76.7	(69.1,82.9)	125	68.0	(52.9,80.0)	0.07
Secondary	90	17.9	(13.6,23.3)	37	20.1	(11.9,31.7)	
≥ Tertiary	27	5.4	(2.2,12.6)	22	12.0	(6.5,21.0)	
Employment status							
Employed	63	12.4	(8.4,18.0)	23	12.3	(6.8,21.2)	0.09
Self-employed	361	71.6	(64.7,77.6)	119	64.6	(53.7,74.2)	
Unemployed/retired	81	16.0	(12.1,20.8)	43	23.1	(16.7,31.0)	
Monthly income (US Dollar)							
≤50	252	49.9	(43.0,56.8)	75	40.4	(30.4,51.2)	0.07
>50	253	50.1	(43.2,57.0)	110	59.6	(48.8,69.6)	
Food security							
Secure	140	27.8	(23.0,33.2)	52	28.1	(21.2,36.3)	0.94
Insecure	364	72.2	(66.8,77.0)	133	71.9	(63.7,78.8)	
**Behavioral and psychological characteristics**							
Alcohol use							
Never	246	48.9	(41.6,56.2)	108	58.5	(48.3,68.0)	0.01
≤Few times per month	147	29.2	(23.1,36.2)	58	31.2	(22.5,41.5)	
≥Few times per week or daily	111	21.9	(17.6,27.0)	19	10.3	(6.0,17.2)	
Smoking history							
Never	362	71.8	(66.7,76.4)	162	88.0	(81.4,92.5)	<0.01
Former smoker	69	13.7	(10.8,17.3)	16	8.9	(5.3,14.5)	
Current smoker	73	14.5	(11.1,18.7)	6	3.1	(1.3,7.3)	
Fruit and vegetable intake (fruits ≥once a week, vegetables daily)							
Neither	79	15.6	(12.2,19.7)	23	12.3	(6.7,21.5)	0.09
Either	244	48.4	(42.9,53.8)	76	40.9	(32.3,50.2)	
Both	182	36.0	(29.7,43.0)	86	46.8	(35.8,58.1)	
Physical activity (activities of daily life and sports ≥once a week)							
Neither	38	7.5	(4.8,11.7)	16	8.9	(5.6,14.0)	0.01
Either	350	69.2	(63.4,74.5)	148	79.9	(72.0,86.1)	
Both	117	23.2	(18.6,28.6)	21	11.1	(6.6,18.1)	
Salt intake (g/day) ^a^							
<5	370	73.3	(69.1,77.1)	153	83.0	(75.0,88.8)	0.06
≥5	120	23.7	(19.6,28.4)	29	15.6	(9.9,23.8)	
Don't know	15	3.0	(1.5,5.8)	3	1.4	(0.3,5.8)	
Psychological distress							
Low	398	78.9	(74.5,82.8)	162	88.0	(81.8,92.3)	0.01
High	106	21.1	(17.2,25.5)	22	12.0	(7.7,18.2)	
**Clinical characteristics**							
Blood pressure ^b^							
Normal	338	66.9	(62.2,71.3)	100	54.0	(43.8,64.0)	0.01
High	167	33.1	(28.7,37.8)	85	46.0	(36.0,56.2)	
Waist-to-hip ratio							
<0.85	369	73.1	(67.7,77.9)	75	40.7	(32.5,49.3)	<0.01
0.85–0.89	96	18.9	(15.3,23.2)	61	32.9	(25.4,41.4)	
≥0.90	40	7.9	(5.7,10.9)	49	26.5	(19.0,35.6)	
HbA1c (%)							
<5.7	322	63.9	(56.6,70.6)	82	44.6	(36.6,52.8)	<0.01
5.7–6.4	177	35.1	(28.4,42.5)	89	48.5	(40.4,56.6)	
≥6.5	5	1.0	(0.4,2.8)	13	7.0	(4.1,11.7)	
Blood lipid profile ^a^^,^^c^							
Normal	96	18.9	(14.6,24.2)	16	8.7	(5.6,13.1)	<0.01
Abnormal	405	80.1	(74.7,84.6)	168	91.3	(86.9,94.4)	
Missing data	5	0.9	(0.3,2.7)	0	0		
**Medical history (self-reported)**							
Hypertension	31	6.2	(4.1,9.4)	24	12.8	(8.3,19.2)	<0.01
Diabetes	1	0.2	(0.0,1.5)	4	2.2	(0.9,5.6)	0.01
HIV infection	60	11.9	(8.8,15.9)	11	6.1	(3.4,10.9)	0.05

Percentages might not sum to 100 due to rounding.

Some discrepancies in the numbers are also due to rounding.

CI: confidence interval

BMI: body mass index

^a^. "Missing data” and "Don't know" were excluded from statistical testing.

^b^. High blood pressure is defined as systolic blood pressure ≥140 mmHg or diastolic blood pressure ≥90 mmHg.

^c^. Abnormal blood lipid includes any abnormal measurements in total cholesterol (≥220 mg/dl), LDL-cholesterol (≥140 mg/dl), HDL-cholesterol (≤40 mg/dl), or triglycerides (≥150 mg/dl).

Table 3 shows the results from the multiple logistic regression analysis overall and by gender. In terms of socio-demographic variables, female gender (adjusted odds ratio (AOR), 2.45; 95% CI, 1.52–3.97), age 45–64 years (AOR, 2.38; 95% CI, 1.58–3.61), secondary education (AOR, 1.75; 95% CI, 1.06–2.90), tertiary education (AOR, 3.25; 95% CI, 1.56–6.77), current smoker (AOR, 0.29; 95% CI, 0.11–0.78), and eating both fruits and vegetables daily (AOR, 1.97; 95% CI, 1.05–3.68) were significantly associated with BMI ≥25 kg/m^2^. Participants with high blood pressure (AOR, 1.79; 95% CI, 1.20–2.68), HbA1c ≥5.7% (AOR, 1.80; 95% CI, 1.22–2.67), abnormal blood lipid profile (AOR, 1.88; 95% CI, 1.04–3.40) also had increased odds of having BMI ≥25 kg/m^2^. In contrast, psychological distress (AOR, 0.40; 95% CI, 0.23–0.68) was associated with lower odds of having BMI ≥25 kg/m^2^. Monthly income, alcohol use, and HIV infection were not associated with having BMI ≥25 kg/m^2^.

**Table 3:** Multivariate correlates of BMI ≥25 kg/m^2^ in a rural district of Zambia.

	Male			Female			Total		
	AOR (95% CI)		*p* value	AOR (95% CI)		*p* value	AOR (95% CI)		*p* value
Gender									
Male	−	−	−	−	−	−	1	(Reference)	
Female							2.39	(1.47–3.88)	<0.01
Age group (year)									
25–44	1	(Reference)		1	(Reference)		1	(Reference)	
45–64	4.19	1.96–9.00	<0.01	1.80	(1.06–3.06)	0.03	2.34	(1.54–3.56)	<0.01
Education level									
≤Primary	1	(Reference)		1	(Reference)		1	(Reference)	
Secondary	1.34	(0.59–3.07)	0.49	1.75	(0.87–3.55)	0.12	1.77	(1.06–2.94)	0.03
≥Tertiary	3.09	(0.96–10.00)	0.06	3.68	(1.23–10.85)	0.02	3.41	(1.61–7.23)	<0.01
Monthly income (US Dollar)									
≤50	1	(Reference)		1	(Reference)		1	(Reference)	
>50	1.46	(0.68–3.13)	0.33	1.13	(0.69–1.87)	0.62	1.20	(0.80–1.81)	0.37
Smoking history									
Never	1	(Reference)		1	(Reference)		1	(Reference)	
Former smoker	1.05	(0.44–2.48)	0.92	0.35	(0.06–2.00)	0.24	0.78	(0.38–1.59)	0.49
Current smoker	0.32	(0.10–1.00)	0.05	0.29	(0.02–3.81)	0.35	0.29	(0.11–0.78)	0.01
Alcohol use									
Never	1	(Reference)		1	(Reference)		1	(Reference)	
≤Few times per month	0.97	(0.41–2.32)	0.94	1.69	(0.93–3.08)	0.08	1.35	(0.84–2.17)	0.22
≥Few times per week or daily	0.77	(0.28–2.12)	0.61	1.11	(0.39–3.17)	0.84	0.90	(0.46–1.75)	0.75
Fruit and vegetable intake (fruits ≥once a week, vegetables daily)									
Neither	1	(Reference)		1	(Reference)		1	(Reference)	
Either	1.02	(0.36–2.94)	0.97	1.33	(0.60–2.95)	0.48	1.19	(0.64–2.21)	0.59
Both	1.42	(0.49–4.15)	0.52	2.44	(1.10–5.41)	0.03	2.00	(1.07–3.75)	0.03
Physical activity (activities of daily life and sports ≥once a week)									
Neither	1	(Reference)		1	(Reference)		1	(Reference)	
Either	0.33	(0.09–1.23)	0.10	1.65	(0.74–3.68)	0.22	1.14	(0.59–2.22)	0.69
Both	0.16	(0.03–0.70)	0.02	1.14	(0.35–3.67)	0.83	0.56	(0.23–1.33)	0.18
Salt intake (g/day)									
<5	1	(Reference)		1	(Reference)		1	(Reference)	
≥5	1.01	(0.45–2.29)	0.97	0.84	(0.44–1.61)	0.61	0.88	(0.54–1.44)	0.61
Psychological distress									
Low	1	(Reference)		1	(Reference)		1	(Reference)	
High	0.72	(0.28–1.84)	0.49	0.31	(0.16–0.60)	<0.01	0.40	(0.23–0.69)	<0.01
Blood pressure									
Normal	1	(Reference)		1	(Reference)		1	(Reference)	
High	1.66	(0.83–3.33)	0.16	1.85	(1.10–3.13)	0.02	1.78	(1.19–2.68)	0.01
HbA1c (%)									
<5.7	1	(Reference)		1	(Reference)		1	(Reference)	
≥5.7	1.55	(0.74–3.26)	0.25	1.80	(1.10–2.95)	0.02	1.72	(1.16–2.55)	0.01
Abnormal blood lipid profile									
No	1	(Reference)		1	(Reference)		1	(Reference)	
Yes	3.16	(1.08–9.29)	0.04	1.50	(0.69–3.27)	0.30	1.86	(1.02–3.37)	0.04
HIV status (self-reported)									
Negative	1	(Reference)		1	(Reference)		1	(Reference)	
Positive	1.02	(0.30–3.48)	0.98	0.70	(0.32–1.57)	0.39	0.76	(0.39–1.48)	0.42

CI: confidence interval

AOR: adjusted odds ratio

In terms of gender differences, we found that abnormal blood lipid profile (AOR, 3.04; 95% CI, 1.05–8.84), and physical activity involving both daily life activities and sports (AOR, 0.18; 95% CI, 0.04–0.77) were significantly associated with BMI ≥25 kg/m^2^ only in men. There was also trend toward significance for current smoker (AOR, 0.32; 95% CI, 0.10–1.00). In women, eating both vegetables daily and fruits more than once a week (AOR, 2.47; 95% CI, 1.12–5.47) and psychological distress (AOR, 0.30; 95% CI, 0.15–0.59) were significant. All other variables were associated with BMI ≥25 kg/m^2^ in similar magnitudes and directions in both genders, except for age 45–64 years, which was more strongly associated with BMI≥25 kg/m^2^ in men versus women.

**Fig 2:**
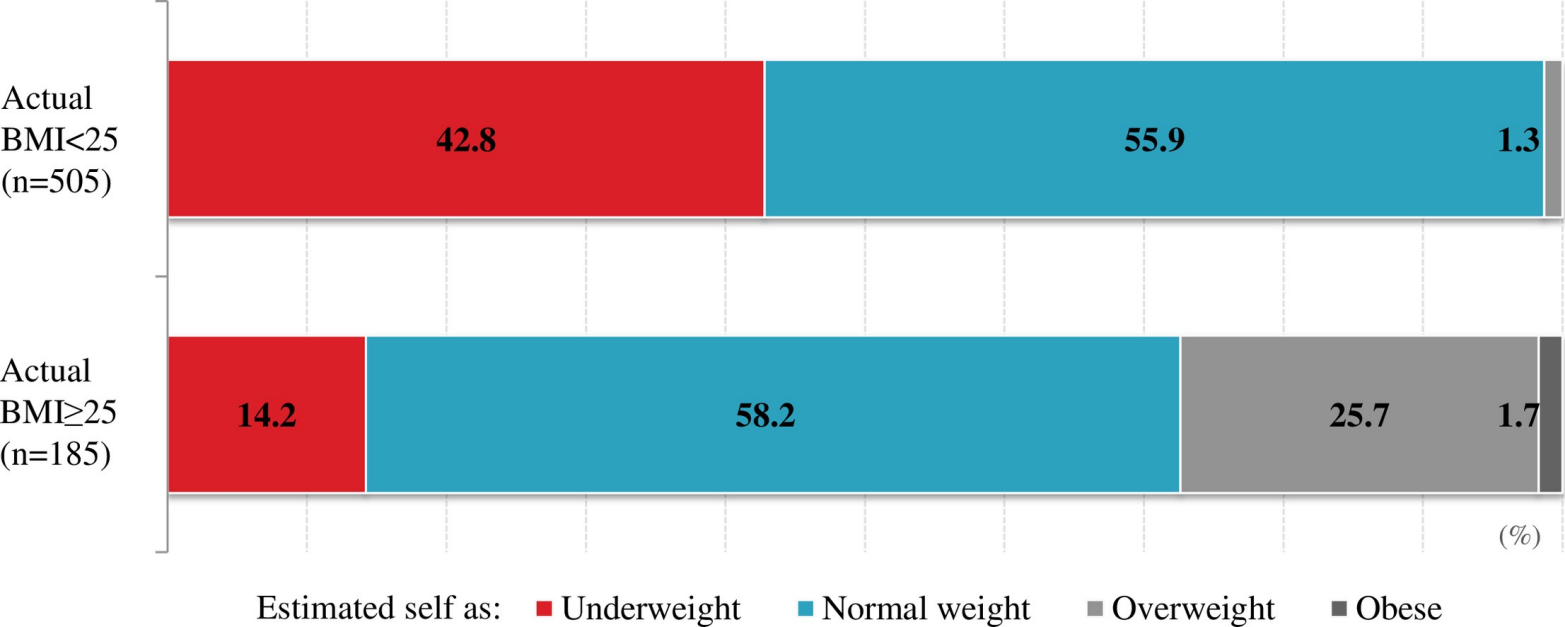
Estimation of body weight by BMI.

Regarding weight perception, 58.2% of participants with BMI ≥25 kg/m^2^ perceived their body weight as normal, while 14.2% perceived themselves as being underweight (Fig 2). The level of agreement between measured body weight and perception of body weight was low (kappa = 0.07). Regarding preference for being overweight, 17.5% of participants with BMI <25 kg/m^2^ and 3.6% of participants BMI ≥25 kg/m^2^ expressed a preference for being overweight. The main reasons for preferring to be overweight included "looks attractive" and "culture/tradition" (n = 74); "looks wealthy" or "avoid to be seen poor" (n = 48); and "looks healthy" (n = 7). Another reason included "scared of being seen to be sick" (n = 32), of them, 31 participants (96.8%) reported "fear of being perceived as HIV-positive".

Table 4 shows the bivariate and multivariate correlates of body weight underestimation among participants with BMI≥25 kg/m^2^. Age was the only variable significantly associated with body weight underestimation; participants aged 45–64 years were significantly more likely to underestimate their body weight compared to participants in younger age groups (AOR, 2.25; 95% CI, 1.13–4.49).

**Table 4:** Multivariate correlates of body weight underestimation among overweight and obesity participants in a rural district of Zambia.

	Correctly estimated		Under estimated					
	n	% (95% CI)	n	% (95% CI)	*p* value	AOR (95% CI)		*p* value
Gender								
Male	15	29.6 (16.4,47.4)	40	29.6 (22.0,38.5)	0.998	1	(Reference)	
Female	35	70.4 (52.6,83.6)	94	70.4 (61.5,78.0)		1.29	(0.60–2.80)	0.52
Age group (year)								
25–44	33	65.3 (46.9,80.0)	57	42.7 (32.3,53.8)	0.02	1	(Reference)	
45–64	17	34.7 (20.0,53.1)	77	57.3 (46.2,67.7)		2.25	(1.13–4.49)	0.02
Education level								
Primary	31	62.2 (39.2,80.9)	94	70.4 (55.5,81.9)	0.39	1	(Reference)	
≥Secondary	19	37.8 (19.1,60.8)	40	29.6 (18.1,44.5)		1.2	(0.53–2.75)	0.66
Employment status								
Employed	10	19.4 (7.9,40.2)	13	9.6 (4.7,18.7)	0.053	1	(Reference)	
Self-employed	24	48.0 (30.5,66.0)	95	70.8 (60.5,79.3)		2.38	(0.82–6.90)	0.11
Unemployed/retired	17	32.7 (20.7,47.4)	26	19.6 (13.4,27.8)		1.04	(0.36–3.03)	0.94

Percentages might not sum to 100 due to rounding.

Some discrepancies in the numbers are also due to rounding.

CI: confidence interval

AOR: adjusted odds ratio

## Discussion

This is one of the first population-based studies exploring socio-cultural and bio-behavioral risk factors for CVD in rural Zambia. Based on the results of our prior qualitative study in the same community, this study sought to estimate the prevalence of overweight and obesity, analyze the socio-cultural and bio-behavioral correlates of overweight and obesity, and evaluate the degree and correlates of body weight underestimation, in order to inform future CVD prevention programs in Zambia. This study found that overweight and obesity were prevalent among the targeted rural community of Zambia, were significantly associated with cardio-metabolic disorders but were largely underestimated by the participants.

### Prevalence of overweight and obesity

We found that 26.8% of participants had BMI ≥25 kg/m^2^; this proportion was much higher in women (36.7%) than in men (16.1%). The overall prevalence of 26.8% is close to the national estimates by WHO in 2010 (26.4%), 2014 (29.2%), and 2017 (24.2%) [10,28], but much higher than in other rural districts such as Kaoma (9.8%) and Kasama (10.3%), which were surveyed in 2008–2009 [17]. However, the prevalence of 26.8% was lower than in the Lusaka (39.3%) and Kitwe (41.6%) urban districts, surveyed in 2007 and 2011, respectively [18,24]. Considering the fact that our study area, Mumbwa, is only 150 km away from the capital Lusaka and that Kaoma and Kasama are remote rural districts, our study site might be a rural area undergoing especially rapid urbanization, or the study site reflects a newer trend of urbanization across all rural region of Zambia. Periodic nationwide surveys of body weight in Zambia are clearly needed to monitor national and regional trends in order to focus prevention programs.

In addition, the nearly two-fold male-to-female difference in the prevalence of overweight and obesity is noteworthy. Such a gender difference has been commonly observed in African regions [10]; this phenomenon is closely related to the socio-cultural context described below. Among our participants, the majority (60%) of overweight and obesity was central (waist-to-hip ratio ≥0.85), which has been well documented to be associated with an elevated risk of CVD [27].

### Demographic correlates of overweight and obesity

Among demographic factors, in both genders, higher age group and higher education level were associated with an increased risk of overweight and obesity, while monthly income showed no association.

An increased risk of overweight and obesity in older age groups and women is well established in SSA, including Zambia [4,29]. The large gender gap in overweight and obesity is particularly noteworthy because it suggests that the life course of excessive weight gain differs by gender: it starts in middle age in men but begins much earlier in women, probably reflecting the fact that obesity has been culturally accepted or even considered desirable, particularly in women. A similar gender difference has been reported in other SSA countries [30–32]. This observation strongly suggests that intensive intervention programs based on best practices for preventing overweight or obesity should be taken in consideration given such a gender gap; they should start in adolescence for women, but before or during middle age for men.

The association between higher education and overweight and obesity has also been well established in many SSA countries [30–33], but is contrary to findings in industrialized countries and Latin American countries, where overweight and obesity are more concentrated among people with lower education levels [33–35]. This might be a transitory phenomenon among SSA countries experiencing recent economic growth in which a Westernized lifestyle is first adopted by populations with more education [30].

The lack of an association between monthly income and overweight and obesity was unexpected because it is a well-established risk factor for overweight and obesity in many parts of the world [36]. The effect of monthly income might have been attenuated by the presence of education level in the logistic model. Another possible explanation is that in rural contexts, household income does not necessarily reflect access to food. Many studies have indicated multiple ways of accessing foods in rural settings, including home farming and community social supports [37]. During our prior qualitative field study, we observed that local people were generally able to access food, especially vegetables, through home farming or at costs that are affordable even for low-income families from neighbors or at the market.

### Lifestyle correlates of overweight and obesity

Our study showed that a higher frequency of fruit and vegetable intake is associated with overweight and obesity only in women, contrary to the well-known negative association between fruit and vegetable intake and body weight in both genders observed in developed countries and increasingly in low- and middle-income settings outside of SSA. Although a clear explanation is not possible, it might be due to the limitations of our questionnaire, which asked about fruit and vegetable intake only in terms of frequency, not quantity. In addition, women might have consumed more fruits and vegetables relative to their level of physical activity. In the prior qualitative study, we observed that vegetables were always cooked with a lot of fat or salad oil, and a family consumed an entire 2.5 L bottle of salad oil each week (unpublished observations). Further studies are clearly needed to assess caloric intake in both men and women in relation to their physical activity levels to inform appropriate CVD prevention programs among Zambians.

Smoking, psychological distress, alcohol use, and physical inactivity are well established risk factors for CVD in SSA and industrial countries [24]. The prevalence of current smoking was limited in our study population, 14.5% in men and 3.1% in women. Current smoking was negatively associated with body weight in the overall sample. However, this association was not statistically significant in female, although there was a trend toward significance in the male sample (*p* value: 0.051). This finding confirms the well-known relationship between smoking and body weight, probably mediated by the metabolic effects of nicotine [38]. Smoking prevalence should be carefully monitored since smoking increases insulin resistance and is associated with central fat accumulation [39,40], which is already prevalent in our study population and increases the risk of CVD. Psychological distress may lead to either weight gain or weight loss [41,42]. Our data showed that women with high levels of psychological distress are less likely to be overweight or obese. In the SSA context, including Zambia, although future study is needed, women might have more stressful lives than men because of their lower social, cultural, and economic status at home and in society, which limits their access to sufficient amounts of food [43,44].

Previous studies in SSA have reported that alcohol consumption is not related or negatively associated with obesity [6,17,45,46], suggesting that alcohol consumption might play at most a minor role, in overweight or obesity in the current SSA context, including Zambia. However, it should be noted again that we only measured the frequency but not the actual amount of alcohol consumed. More accurate assessment of alcohol consumption would be needed to inform future intervention programs for promoting healthy lifestyles. Regarding the association of physical activities with overweight and obesity, the gender difference observed in our study is consistent with findings from other studies in SSA, including Zambia [6]. This discordance has been explained by the fact that males walk more in their day-to-day life or have heavier workloads than females in these social settings [6].

### Cardio-metabolic correlates of overweight and obesity

Most of the biomarkers measured in our study were clearly associated with overweight and obesity. People who were overweight or obese were more likely to have elevated blood pressure and HbA1c, and abnormal blood lipid profiles compared to those with normal body weight. The association between high blood pressure and obesity is well documented in SSA countries, including both urban and rural areas of Zambia [6,17]. Although the relationship of blood glucose and blood lipid profile with obesity is well established in industrialized countries, as well as in SSA [5,47–49], our findings are the first in Zambia. Given the potential for continued lifestyle changes Zambia in the future, overweight and obesity might increase over time, potentially leading to an increase in the prevalence of hypertension, hyperglycemia, and hyperlipidemia and eventually the incidence of CVDs.

### Attitudes toward being overweight

Our study showed that 17.5% of the BMI <25 kg/m^2^ group preferred being overweight, compared with 3.6% of the BMI ≥25 kg/m^2^ group and 13.8% overall. Of them, nearly 80% reported “looks attractive” as a reason for preferring being overweight. The presence of such a preference for being overweight has been consistently reported in SSA countries [12,50–52]. It is assumed to be a SSA-specific cultural preference, linked with social success, happiness, and wealth in poor settings [12,16,50,53]. Attitudes toward being overweight were found to be partially affected by the HIV epidemic, confirming the results of our prior qualitative study. Our study showed that 30 of 95 subjects (31.6%) who preferred being overweight cited "fear of being perceived as HIV-positive" as the reason. Similar attitudes about body weight associated with HIV stigma have also been reported in other SSA countries [12,14,16,30,54–56]. Although it affected less than 5% of all participants, future CVD prevention programs in countries hit hard by the HIV epidemic should carefully consider this point.

### Underestimation of body weight

A particularly noteworthy finding of our study is that many overweight and obese subjects were not aware that they are physically overweight and obese. In fact, among participants with BMI ≥25 kg/m^2^, 14.2% and 58.2% perceived themselves as underweight and normal weight, respectively. This may be due to their perception of standard size being higher because of the cultural preference for obesity. Very limited opportunities to actually measure body weight might contribute to misperceptions about body weight. Underestimation of body weight has been recently reported in other LMICs such as Sri Lanka, Pakistan, and Nigeria [57–59].

In our study, higher age (44–64 years) was associated with more underestimation, which is consistent with findings from studies in Sri Lanka and Pakistan [58,59]. This age difference may suggest that younger individuals have more opportunities to be aware of their own body weight compared to older individuals [58,59]. However, our finding contradicts with findings in Nigeria, where the underestimation of weight was associated with being in younger age group, female gender, and employment status [57]. Whatever the pattern of association, given the fundamental importance of body weight self-awareness in CVD prevention programs, it is important to understand how such a misperception of body weight arose in the SSA context.

### Strengths and limitations

Our study has several strengths and limitations. The strengths of this study include the sequential mixed methods design, which allowed our prior qualitative findings to be successfully reinforced by quantitative findings. In addition, this study employed a multi-stage cluster random sampling with a high response rate, which helps ensure that the results are representative of the residents in the study area. In addition, our study is the first to introduce biomarker measurements (HbA1c, LDL-/ HDL-cholesterol, and urinary-Na/K ratio) to investigations of CVD risk factors in Zambia. On the other hand, limitations of our study include the fact that approximately one-third of the participants failed to fast before testing despite our instructions. This might have affected biological measurements, particularly of blood sugar and lipids, although actually there were no or large differences between fasting and non-fasting subjects in terms of biological parameters. In addition, socially desirable answers could have affected the results, since we employed face-to-face interviews with trained interviewers rather than self-administered questionnaires. However, face-to-face interviewing was the only data collection method considered given the low literacy rates among the study population. Finally, some of the associations found in this study may be confounded by unmeasured factors, even though we tried to include all possible variables from previous studies.

## Conclusions

Our study revealed that more than one-fourth of the respondents were overweight or obese in a rural district of Zambia. Overweight and obesity are significantly associated with lifestyle-related risk factors for CVD and other cardio-metabolic disorders. More importantly, we found that one-fifth of our sample prefers overweight and that the majority (>70%) of overweight or obese individuals do not perceive themselves as being overweight or obese. These results suggest that overweight and obesity are already having substantial impact on the health of the people in rural Zambia, although their roles have not been recognized. Weight control programs should be urgently introduced to prevent increase of CVDs, with a particular focus on the correction of body weight underestimation.

## Supporting information

S1 Appendix Questionnaire (English). (PDF)

S2 Appendix Questionnaire (Local languages). (PDF)
