# Supplementary material for: Obesity matters but is not perceived: A cross-sectional study on cardiovascular disease risk factors among a population-based probability sample in rural Zambia
Source: PLoS One. 2018 Nov 29;13(11):e0208176. doi: 10.1371/journal.pone.0208176 (PMC6264511; doi:10.1371/journal.pone.0208176)
Supplement: S1 Appendix — (PDF) [file pone.0208176.s001.pdf]

Socio-epidemiological study on risk factors of non-communicable diseases among adult population in rural Zambia

LOCATION AND DATE		
A.	Cluster number	└─┴─┴─┘
B.	Household number	└─┴─┴─┘
C.	Name and code of Village/compound	....
D.	Residential Classification	1: High Density<br>2: Low Density
E.	Language for interview	1: English<br>2: Bemba<br>3: Nyanja

VISIT RECORD				
	**1**	**2**	**3**	**Final visit**
**Interview Date**	(Day/Month)	(Day/Month)	(Day/Month)	(Day/Month)
**Physical/Biomedical Measurement Date**	(Day/Month)	(Day/Month)	(Day/Month)	(Day/Month)
**Interviewer's Name**				
**Result***				
***Result Codes**	1. Completed<br>2. Postponed<br>3. Refused<br>4. Household absent<br>5. Participants absent<br>6. Other(specify)			

CRITERIA			
001	How long have you lived in this village/town?		________ months/years<br>Less than 6 months →not eligible
002	**For women:**<br>Are you pregnant?<br>Do you have a baby less than 6 months?	Yes<br>No	1<br>2
003	Is your age ***between 25 and 64 years old?***	Yes<br>No	1<br>2

Please circle the eligibility of this study

Eligible / Not Eligible

Physical Measurements

Blood Pressure

※*Make sure that participants have already seated calmly for* ***at least 15 minutes***

During the past two weeks, have you been treated for raised blood pressure with drugs (medication) prescribed by a doctor or other health worker?	Yes<br>No	1<br>2

Cuff size used

1: Small
2: Medium
3: Large

(mmHg)	Systolic	Diastolic
Reading 1	└─┴─┴─┘	└─┴─┴─┘
Reading 2	└─┴─┴─┘	└─┴─┴─┘
Reading 3	└─┴─┴─┘	└─┴─┴─┘

Height, Weight, Waist and Hip Circumference

Height	cm	└─┴─┴─┘ . └─┘	Waist circumference	cm	└─┴─┴─┘ . └─┘
Weight	kg	└─┴─┴─┘ . └─┘	Hip circumference	cm	└─┴─┴─┘ . └─┘

Biochemical Measurements

Since 8 pm yesterday, have you had ***anything to eat or drink***, other than water?	Yes<br>No	1<br>2
Today, have you **taken insulin or medications** prescribed by a doctor for ***raised blood glucose***?	Yes<br>No	1<br>2
During the past two weeks, have you been **taken medications** prescribed by a doctor for ***raised cholesterol***?	Yes<br>No	1<br>2

Blood test (Glucose/ Lipids)

Time of day blood specimen taken (24 hour clock)			Hours : minutes		└─┴─┘ : └─┴─┘
Fasting blood glucose	mg/dl	└─┴─┴─┘ . └─┘	Triglycerides	mg/dl	└─┴─┴─┘ . └─┘
HbA1c	%	└─┴─┘ . └─┘	LDL-Cholesterol	mg/dl	└─┴─┴─┘ . └─┘
Total cholesterol	mg/dl	└─┴─┴─┘ . └─┘	HDL Cholesterol	mg/dl	└─┴─┴─┘ . └─┘

Urine test (Urinary Na/K ratio and urine sugar)

Time of day blood specimen taken (24 hour clock)		Hours : minutes	└─┴─┘ : └─┴─┘
Urinary Na/K ratio	└─┴─┘ . └─┘	Urinary sugar	1: (－)<br>2: (±)<br>3: (＋)<br>4: (2＋)<br>5: (3＋)

S1 Appendix (Questionnaire: English)

Part1: About yourself (14 questions)

We are going to ask about yourself and your family.

101.	What is your Gender?	1<br>2	Male<br>Female
102.	How old are you?		( ) years old
103.	Which ethnic groups do you belong to?	1<br>2<br>3<br>4<br>5	Nyanja/Chewa/Ngoni/Tumbka/Nsenga<br>Bemba<br>Tonga<br>Lozi<br>Others (Please specify) ( )
104.	What is your marital status?	1<br>2<br>3<br>4<br>5<br>6	Never Married<br>Married<br>Married Living Separately<br>Divorced<br>Widow/Widowed<br>Never Married but Living Together
105.	How many people are living in your household?		( ) People
106.	How many people older than 15 years including yourself, live in your household?		( ) People
107.	In your household, who is handling the family budget?	1<br>2<br>3	Myself<br>Husband/Wife<br>Other Family Member
108.	What is the highest level of education you have completed so far?	1<br>2<br>3<br>4<br>5	No school at all<br>Basic school<br>Secondary school<br>College<br>More than College
109.	Which of the following best describes your main work status over the past 6 months?	1<br>2<br>3<br>4<br>5<br>6<br>7	Government employed<br>Non-government employed<br>Self-employed (Self-business)<br>Student<br>Housewife/Househusband<br>Retired<br>Unemployed
110.	How much is your household income per month?	1<br>2<br>3<br>4<br>5<br>6	Less than 500 Kwacha<br>500~999 Kwacha<br>1,000~1,999 Kwacha<br>2,000~2,999 Kwacha<br>More than 3,000 Kwacha<br>Don’t know
111.	111-1. Are you taking any medication?	1<br>2	No<br>**Yes**
	⇒ 111-2. If Yes, what kind of medication? (Please specify your medication)		↳ **( )**<br><br>or 1. Hypertensive drug<br>2. Diabetic drug<br>3. ART (HIV treatment)<br>4. TB drug<br>5. Pain killer
112.	112-1. Are you suffering from any diseases?	1<br>2	No<br>**Yes**
	⇒ 112-2. If Yes, what kind of diseases?		↳ **( )**
**For Women Only**			
113.	How many times were you Pregnant (Gravida) and Delivered (Parity)?		Pregnant (Gravida) ( )<br>Delivery (Parity) ( )
114.	114-1. Are you using any family planning method?	1<br>2	No<br>**Yes**
	⇒ 114-2. If Yes, What kind of family planning method are you using?		↳ **( )**

Part2: Food Security (9 questions)

We are going to ask about food issues in your household.

201.	201-1. In the past 30 days, did you worry that your household would not have enough food?	1<br>2	No<br>Yes →
	⇒ 201-2. If Yes, how often did this happen in the past 30 days?	1<br>2<br>3	Rarely (1-2 times)<br>Sometimes (3-10 times)<br>Often (more than 10 times)
202.	202-1. In the past 30 days, were you or any household member not able to eat the kinds of foods you preferred because of a lack of resources?	1<br>2	No<br>Yes →
	⇒ 202-2. If Yes, how often did this happen in the past 30 days?	1<br>2<br>3	Rarely (1-2 times)<br>Sometimes (3-10 times)<br>Often (more than 10 times)
203.	203-1. In the past 30 days, did you or any household member have to eat a limited variety of foods due to a lack of resources?	1<br>2	No<br>Yes →
	⇒ 203-2. If Yes, how often did this happen in the past 30 days?	1<br>2<br>3	Rarely (1-2 times)<br>Sometimes (3-10 times)<br>Often (more than 10 times)
204.	204-1. In the past 30 days, did you or any household member have to eat some foods that you really did not want to eat because of a lack of resources to obtain other types of food?	1<br>2	No<br>Yes →
	⇒ 204-2. If Yes, how often did this happen in the past 30 days?	1<br>2<br>3	Rarely (1-2 times)<br>Sometimes (3-10 times)<br>Often (more than 10 times)
205.	205-1. In the past 30 days, did you or any household member have to eat a smaller meal than you felt you needed because there was not enough food?	1<br>2	No<br>Yes →
	⇒ 205-2. If Yes, how often did this happen in the past 30 days?	1<br>2<br>3	Rarely (1-2 times)<br>Sometimes (3-10 times)<br>Often (more than 10 times)
206.	206-1. In the past 30 days, did you or any household member have to eat fewer meals in a day because there was not enough food?	1<br>2	No<br>Yes →
	⇒ 206-2. If Yes, how often did this happen in the past 30 days?	1<br>2<br>3	Rarely (1-2 times)<br>Sometimes (3-10 times)<br>Often (more than 10 times)
207.	207-1. In the past 30 days, was there ever no food to eat of any kind in your house because of lack of resources to get food?	1<br>2	No<br>Yes →
	⇒ 207-2. If Yes, how often did this happen in the past 30 days?	1<br>2<br>3	Rarely (1-2 times)<br>Sometimes (3-10 times)<br>Often (more than 10 times)
208.	208-1. In the past 30 days, did you or any household member go to sleep at night hungry because there was not enough food?	1<br>2	No<br>Yes →
	⇒ 208-2. If Yes, how often did this happen in the past 30 days?	1<br>2<br>3	Rarely (1-2 times)<br>Sometimes (3-10 times)<br>Often (more than 10 times)
209.	209-1. In the past 30 days, did you or any household member go a whole day and night without eating anything because there was not enough food?	1<br>2	No<br>Yes →
	⇒ 209-2. If Yes, how often did this happen in the past 30 days?	1<br>2<br>3	Rarely (1-2 times)<br>Sometimes (3-10 times)<br>Often (more than 10 times)

Part3: Lifestyle (9 questions)

We are going to ask about your lifestyle and dietary habits.

Tobacco Use			
301.	301-1. Have you ever smoked tobacco?	1<br>**2**	No ⇒ Skip to 402<br>**Yes**
	⇒ 301-2. If yes, how often do you smoke tobacco?	1<br>2<br>3<br>4	I have tried only a few times before<br>I'm a previous/Ex-smoker<br>I am a current smoker, but not every day<br>**I am currently a daily smoker**
	⇒ 301-3. If answer is 5, how many tobaccos do you smoke per day?		**( )**

Alcohol Use			
302.	302-1. Have you ever drunk any alcohol?	1<br>**2**	No ⇒ Skip to 306<br>**Yes**
	⇒ 302-2. If yes, how often do you drink alcohol?	1<br>2<br>3<br>4<br>5<br>6<br>7	Only a few times before<br>Once a month<br>2-3 times a month<br>1-2 days per week<br>3-4 days per week<br>Nearly everyday<br>Everyday
	⇒ 302-3. For what reasons do you drink alcohol? (Mark all that apply)	1<br>2<br>3<br>4<br>5<br>6	To forget your problems<br>To relax<br>For enjoyment<br>To drink with friends/family<br>To help digestion<br>Others (Please specify) ( )

303~305. What do you think of smoking tobacco and taking alcohol?

	**303. Can affect you and your own health?**		
	1<br>No	2<br>Yes	77<br>Don't know
**Tobacco**			
**Smoking around you**			
**Alcohol**			

	**304. Is it *<u>good</u>* your health?**		
	**1<br>No**	2<br>Yes	77<br>Don't know
**Tobacco**			
**Smoking around you**			
**Alcohol**			

	**305. If *<u>No</u>*, how affect you and your own health?**												
	1<br>Harm your lung	2<br>Harm your liver	3<br>Harm your stomach	4<br>Harm your heart	5<br>Harm your brain	6<br>Harm your kidney	7<br>Harm your vein	8<br>get TB	9<br>get cough	10<br>get family problem	11<br>get poverty	12<br>Others	13<br>Don't know
**Tobacco**													
**Smoking around you**													
**Alcohol**													

Physical activity

306	306-1. Does ***your work*** involve intensity activity that causes ***increases in breathing or heart rate (like carrying or lifting heavy/light loads, digging or construction work, farming, etc.)*** for ***at least 10 minutes*** continuously? *(except commuting time)*	1<br>**2**	No ⇒ Skip to 307<br>**Yes**
	⇒ 306-2. If Yes, how is the activity like?	1<br>2	Vigorous (large workload)<br>Moderate (small workload)
	⇒ 306-3. What kind of activities do you do?		**( )**
	⇒ 306-4. How many days in a typical week?		**( )**Days
	⇒ 306-5. How much time in a typical day?		**( )**hours
307	307-1. Do you do any intensity ***sports, fitness or recreational (leisure) activities*** that cause increases ***in breathing or heart rate (like running or football, cycling, swimming, netball, etc.)*** for at least 10 minutes continuously?	1<br>**2**	No ⇒ Skip to 308<br>**Yes**
	⇒ 307-2. If Yes, what kind of activities do you do?		**( )**
	⇒ 307-3. How many days in a typical week?		**( )**Days
	⇒ 307-4. How much time in a typical day?		**( )**hours
308	308-1. Do you ***walk or use a bicycle*** for ***more than 10 minutes*** continuously to go to work or go somewhere in your daily life?<br>****This questions is focused on transport-related physical activity to travel around getting from place-to-place***	1<br>**2**	No ⇒ Skip to 309<br>**Yes**
	⇒ 308-2. If Yes, how many days in a typical week?		**( )**Days
	⇒ 308-3. How much time in a typical day?		**( )**hours
309	How many hours do you usually ***sitting or reclining*** except regular sleeping time in a typical day?<br>****Consider total time spent at work sitting, in an office, reading, watching television, using a computer, doing hand craft like knitting, resting etc.***		**( )**hours

Part4: Dietary habit (16 questions)

We are going to ask about your dietary habits in your daily meal.

Vegetables and Fruits

※ *In this section, we are asking about* ***green and colored vegetables and fruits*** *(Not potatoes, grains or rice.)*

401.	How many days do you eat **fruits** and **vegetables** in a typical week?		Fruits: **( )** days<br>Vegetables: **( )** days

402. 402-1. Do you have any challenges to eat **fruits** and **vegetables**?

	1<br>No	2<br>Yes	77<br>Don't know
**Fruits**			
**Vegetables**			

⇒ 402-2. If Yes, what are the main reasons of your challenges to eat **fruits** and **vegetables**? (Mark all that apply)

	1<br>Don't have money	2<br>Don't like it	3<br>Don't need it	4<br>Worry chemical use	5<br>Others (Specify)
**Fruits**					
**Vegetables**					

403.	How important is it to you to eat **fruits** and **vegetables** every day?

	1<br>Not important at all	2<br>Not so important	3<br>Moderately important	4<br>Important	5<br>Very important
**Fruits**					
**Vegetables**					

Salad oil and fat

404.	404-1. Do you use oil or fat for your food/ cooking?	1<br>2<br>3	No ⇒ skip to 505<br>**Yes** (→ 404-2)<br>Don't know
	⇒ 404-2. If Yes, What type of fat is most often used for meal preparation in your household? (Mark all that apply)	**1**<br>**2**<br>3<br>4<br>77	**Salad Oil** (→ 404-3)<br>**Butter or Margarine** **(Including Blueband (brand name))** (→ 404-4)<br>Animal Fat (Lard or Suet)<br>Others (Please specify) (**)**<br>Don't Know
	⇒ 404-3. If you answer ***Salad Oil***, how much amount do you or your household use per day on average? *(measure the amount of vegetable oil)*		How many days would it take to finish?<br>☐***750ml*** bottle of salad oil: ( )<br>☐***2.5L*** bottle of salad oil: ( )<br>☐***5L*** bottle of salad oil: ( )<br><br>If you don't you the bottle of salad oil, tell us specific amount per day.<br>☐ ( ) ml<br>☐ ( ) amounts of price
	⇒ 404-4. If you answer ***Butter or Margarine***, how much amount do you or your household use per day on average? *(measure the weight of butter and margarine)*		How many days would it take to finish?<br>☐***250g*** of butter and margarine: ( )

Salt

405.	How often is salt or salty seasoning added in ***cooking or preparing foods*** in your household?	1<br>2<br>3<br>4<br>5<br>77	Never<br>Rarely<br>Sometimes<br>Often<br>Always<br>Don't know
406.	How often do you ***add salt or salty sauce*** on your food (dish) ***before or while you are eating***?	1<br>2<br>3<br>4<br>5<br>77	Never<br>Rarely<br>Sometimes<br>Often<br>Always<br>Don't know
407.	On average, how much salt does your household consume per day?		measure the weight of salt _________ g
408.	408-1. Are you concern of your salt intake?	1<br>**2**<br>3	No ⇒ skip to 409<br>**Yes** (→ 408-2)<br>Don't know
	⇒ 408-2. If yes, do you control your salt intake?	1<br>**2**<br>3	No ⇒ skip to 409<br>**Yes** (→ 408-3)<br>Don't know
	⇒ 408-3. If yes, do you do any of the following on a regular basis to control your salt intake? (Mark all that apply)	1<br>2<br>3<br>4<br>5<br>6<br>7	Reduce the amount of salt intake<br>Reduce processed foods<br>Check the salt content of food labels<br>Buy low salt/sodium foods<br>Use spices other than salt when cooking<br>Avoid eating foods prepared outside of home<br>Others (Please specify) (**)**

S1 Appendix (Questionnaire: English)

**Sugar**			
409.	How often do you add sugar to your food or tea?	1<br>2<br>3<br>4<br>5<br>77	Never<br>Rarely<br>Sometimes<br>Often<br>Always<br>Don't know
410.	How much sugar do you consume per day?		measure the weight of sugar _________ g

411~416. What do you think of your *salad oil, salt and sugar intake*?

	**411. Do you think Zambians take a lot of these?**		
	1<br>No	2<br>Yes	77<br>Don't know
**Salad oil**			
**Salt**			
**Sugar**			

	**412. How much of these do you think you consume?**					
	1<br>Too Much	2<br>A bit too much	3<br>Just the right amount	4<br>A bit too Little	5<br>Too Little	6<br>Don't know
**Salad oil**						
**Salt**						
**Sugar**						

	**413. What is the main reason you take sugar? (Mark all that apply)**					
	1<br>Customs	2<br>Traditions	3<br>Taste	4<br>Health	5<br>Energy	6<br>Others (specify)
**Salad oil**						
**Salt**						
**Sugar**						

	**414. Can affect you and your own health?**		
	1<br>No	2<br>Yes	77<br>Don't know
**Salad oil**			
**Salt**			
**Sugar**			

	**415. Is it good your health?**		
	1<br>No	2<br>Yes	77<br>Don't know
**Salad oil**			
**Salt**			
**Sugar**			

	**416. If No, how affect you and your own health?**												
	1<br>Harm your liver	2<br>Harm your stomach	3<br>Harm your heart	4<br>Harm your brain	5<br>Harm your kidney	6<br>Harm your vein	7<br>get BP	8<br>get diabetes (sugar)	9<br>get over weight	10<br>get stroke	11<br>get heart disease	12<br>Others	13<br>Don't know
**Salad oil**													
**Salt**													
**Sugar**													

S1 Appendix (Questionnaire: English)

Part5: Food Safety (2 questions)

We are going to ask about what you think of the foods in the superstore or market.

501.	501-1. Do you think dietary habits have changed from the past (when you were young)?	1<br>**2**<br>3	No<br>**Yes** → 501-2<br>Don't know
	⇒ 501-2. If yes, what kinds of things have mostly changed ***in the market***? (Mark all that apply)	1<br>2<br>3<br>4<br>5<br>6	Variety of food<br>Price<br>Increase fast-food<br>Increase processed food<br>Increase imported food<br>Others (Please specify) ( )
	⇒ 501-3. What kinds of foods have ***increased*** and ***decreased*** from the past (when you were young?) (Mark all that apply)<br>※ *This question **is not** asking of your availability of food, asking **the food selling in the market.***		

	1<br>Salt	2<br>Sugar	3<br>Salad oil	4<br>Animal fat	5<br>Fish	6<br>Vegetable	7<br>Fruits	8<br>Potatoes	9<br>Beans
**Increased**									
**decreased**									

	10<br>Groundnuts	11<br>Bread	12<br>Snacks	13<br>Juice	14<br>Fast-food	15<br>Processed-food	16<br>Imported food	17<br>Others(specify)
**Increased**								
**decreased**								

502.	502-1. Do you have any concern about foods that are sold in the market?	1<br>**2**<br>3	No<br>**Yes** → 502-2<br>Don't know
	⇒ 502-2. If yes, ***What kind of food*** do you mostly ***concern***? (Mark all that apply)		

1<br>Vegetable	2<br>Fruits	3<br>Chicken	4<br>Beef	5<br>Fish	6<br>Other meats	7<br>Restaurant	8<br>Fast-food	9<br>Selling outside	10<br>Others (specify)

	⇒ 502-3. What are ***your concerns about food***? (Mark all that apply)	1<br>2<br>3<br>4<br>5<br>6	Chemical<br>Injection (to animals)<br>Fertilizer<br>Food quality<br>Food poison<br>Others (Please specify) ( )

Part6: Mental Stress and relationship of neighborhood (12 questions)

We are going to ask about the psychological distress and stress management.

Psychological stress

※These questions concern how you have been feeling *over the past 30 days.*

		None of the time	A little of the time	Some of the time	Most of the time	All of the time
601.	How often did you feel nervous?	1	2	3	4	5
602.	How often did you feel hopeless?	1	2	3	4	5
603.	How often did you feel restless or fidgety?	1	2	3	4	5
604.	How often did you feel depressed that nothing could cheer you up?	1	2	3	4	5
605.	How often did you feel that everything was an effort?	1	2	3	4	5
606.	How often did you feel worthless?	1	2	3	4	5

Stress management			
607.	607-1. Do you feel any stress in your daily life?	1<br>**2**<br>3	No ⇒ skip to 708<br>**Yes**<br>Don’t know
	⇒ 607-2. If Yes, what is currently the main cause of stress in your life? (Mark all that apply)	1<br>2<br>3<br>4<br>5<br>6	Family<br>Relationships<br>School<br>Work<br>Money<br>Health
	⇒ 607-3.What do you do to reduce your stress? (Mark all that apply)		

1<br>Exercise	2<br>Talk to friends/family	3<br>Eat	4<br>Talk to Health workers	5<br>Drink some alcohol	6<br>Pray	7<br>Reading bible	8<br>Sleep	9<br>Don’t do anything	10<br>Others (specify)

Social cohesion and trust						
608.	People around here are willing to help their neighbors	Strongly disagree	Disagree	Neither Agree nor Disagree	Agree	Strongly agree
		1	2	3	4	5
609.	This is a close-knit neighborhood	Strongly disagree	Disagree	Neither Agree nor Disagree	Agree	Strongly agree
		1	2	3	4	5
610.	People in this neighborhood can be trusted	Strongly disagree	Disagree	Neither Agree nor Disagree	Agree	Strongly agree
		1	2	3	4	5
611.	People in this neighborhood generally don't get along with each other	Strongly disagree	Disagree	Neither Agree nor Disagree	Agree	Strongly agree
		1	2	3	4	5
612.	People in this neighborhood do not share the same values	Strongly disagree	Disagree	Neither Agree nor Disagree	Agree	Strongly agree
		1	2	3	4	5

Part7: About overweight/Obesity (8 questions)

We are going to ask about your perception of overweight/Obesity.

Obesity			
701.	What do you think of your body weight?	1<br>2<br>3<br>4	Underweight<br>Normal weight<br>Overweight<br>Very Overweight(Obese)
702.	Do you think Zambians prefer to be overweight?	1<br>2<br>77	No<br>Yes<br>Don’t know
703.	Do you prefer to be overweight?	1<br>**2**<br>77	No<br>**Yes**<br>Don’t know
704.	⇒ 704-1. If yes, what is the main reason why you prefer to be overweight? (Mark all that apply)	1<br>2<br>3<br>4<br>**5**<br>6<br>77	Attractive<br>Looks wealthy<br>Culture/tradition<br>Avoid to be seen poor<br>**Scared of being seen to be sick**<br>Others (Please specify) ( )<br>Don’t know
	⇒ 704-2.If answered 5, what kind of sickness are you scared of being seen??	1<br>2<br>3<br>4<br>5	Tuberculosis<br>Malaria<br>HIV<br>High blood pressure<br>Others (Please specify) ( )

705.	Please see the pictures below.<br>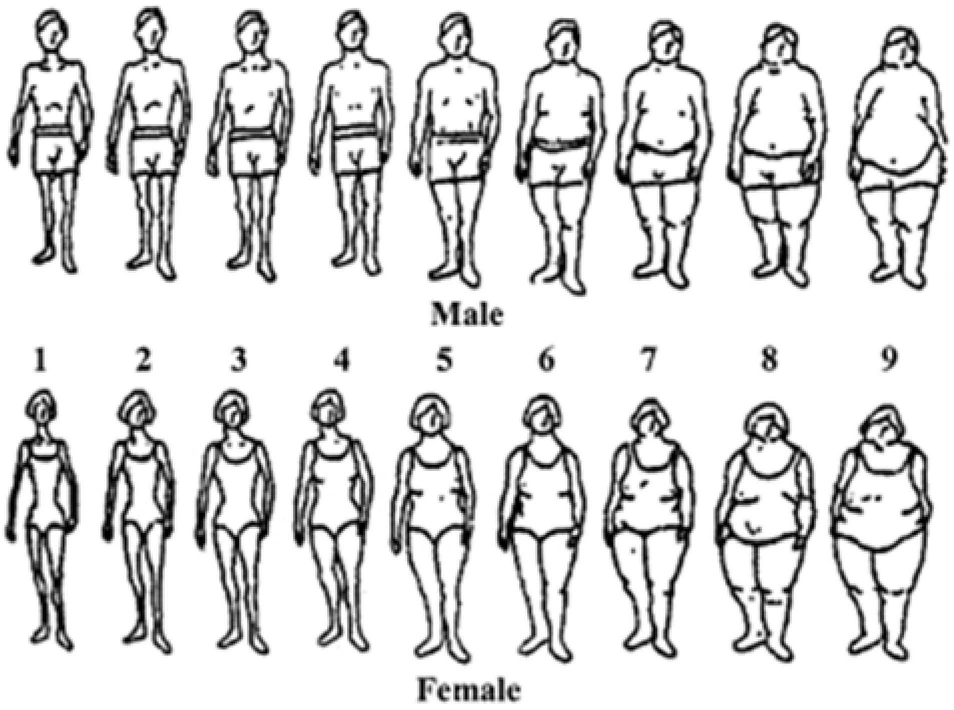		
	705-1. Which number of figure is the closest of your body size?		1 2 3 4 5 6 7 8 9
	705-2. Which number of ***male*** figure do you recognize overweight?		1 2 3 4 5 6 7 8 9
	705-3. Which number of ***female*** figure do you recognize overweight?		1 2 3 4 5 6 7 8 9
706.	How important is having a normal body weight to you?	1<br>2<br>3<br>4<br>5	Not important<br>Not so important<br>Moderately important<br>Important<br>Very important

707~708. What do you think of stress and obesity?

	707. Can these affect you and your own health?		
	1<br>No	2<br>Yes	77<br>Don't know
**Stress**			
**Obesity**			

	708. If Yes, how affect you and your own health?												
	1<br>Harm your liver	2<br>Harm your stomach	3<br>Harm your heart	4<br>Harm your brain	5<br>Harm your kidney	6<br>Harm your vein	7<br>get BP	8<br>get diabetes (sugar)	9<br>get over weight	10<br>get stroke	11<br>get heart disease	12<br>Others	13<br>Don't know
**Stress**													
**Obesity**													

S1 Appendix (Questionnaire: English)

Part8: Cardiovascular diseases (15 questions)

We are going to ask about your knowledge and perception of cardiovascular diseases.

801~811. Please answer the following question?

	801. Do you know the term of following diseases?		
	1<br>No	2<br>Yes	77<br>Don't know
**High BP**			
**Stroke**			
**Heart disease**			
**Diabetes**			

⇒ **If No, or don't know at each disease, skip the questions of each disease.**

	802. Are you concerned about developing these diseases?		
	1<br>No	2<br>Yes	77<br>Don't know
**High BP**			
**Stroke**			
**Heart disease**			
**Diabetes**			

	803. Can these transmit between people?		
	1<br>No	2<br>Yes	77<br>Don't know
**High BP**			
**Stroke**			
**Heart disease**			
**Diabetes**			

	804. Are these dangerous than infectious diseases like HIV?				
	1<br>Strongly disagree	2<br>Disagree	3<br>Neither Agree nor Disagree	4<br>Agree	5<br>Strongly agree
**High BP**					
**Stroke**					
**Heart disease**					
**Diabetes**					

	805. What increase the risk to develop these diseases?											
	1<br>Smoking	2<br>Alcohol	3<br>Stress (thinking a lot)	4<br>Being over-weight	5<br>Getting older	6<br>Too much salt	7<br>Too much oil	8<br>Too much sugar	9<br>Poor diet	10<br>physically inactive	11<br>High BP	77<br>Don't know
**High BP**												
**Stroke**												
**Heart disease**												
**Diabetes**												

	806. Can these diseases be prevented?		
	1<br>No	2<br>Yes	77<br>Don't know
**High BP**			
**Stroke**			
**Heart disease**			
**Diabetes**			

	807. If Yes, how can be prevented?							
	1<br>Improving diet	2<br>Taking medications	3<br>Doing exercise	4<br>Losing weight	5<br>Quit smoking	6<br>Nothing	7<br>Others	77<br>Don't know
**High BP**								
**Stroke**								
**Heart disease**								
**Diabetes**								

	808. Have you ever learned before?		
	1<br>No	2<br>Yes	77<br>Don't know
**High BP**			
**Stroke**			
**Heart disease**			
**Diabetes**			

	809. <u>If Yes</u>, Where did you learn?				
	1<br>Clinic/Hospital	2<br>CHW	3<br>Media(TV, Radio, IT)	4<br>Church	99<br>Others
**High BP**					
**Stroke**					
**Heart disease**					
**Diabetes**					

	810. Are these diseases common in Zambia?		
	1<br>No	2<br>Yes	77<br>Don't know
**High BP**			
**Stroke**			
**Heart disease**			
**Diabetes**			

	811. Is there anyone who has these diseases in your family?		
	1<br>No	2<br>Yes	77<br>Don't know
**High BP**			
**Stroke**			
**Heart disease**			
**Diabetes**			

History of Raised Blood Pressure and Diabetes

812.

812-1. Have you ever had your blood pressure measured?

1 **No**
2 **Yes**

⇒ 812-2. If yes, where did you measure your blood pressure last time?

1<br>Clinic/Hospital	2<br>Workplace	3<br>School	4<br>Home	5<br>Friend's place	6<br>Others

⇒ 812-3. What is the main reason that made you check your blood pressure last time?

1 Regular checkup of hypertension *(already diagnosed high BP)*
2 I had symptoms related to blood pressure
3 Checked blood pressure when I visited clinic for other diseases
4 Health checkup *(not regular checkup)*
5 Others (Please specify) (   )

If No,
⇒ 812-4. What is the main reason that made you not to check your blood pressure before?

1 Don't have time
2 Don't know where to access service
3 Don't think it is important
4 Don't know how
5 Don't have a chance
6 Feel scared to check
7 Others (Please specify) (   )

813.	813-1. Have you ever been told by a doctor or other health worker that you have raised blood pressure or hypertension?	1<br>2<br>77	No<br>**Yes** → 813-2<br>Don't know
	⇒ 813-2. In Yes, have you been told in the past 12 months?	1<br>2	No<br>Yes
814	Have you ever had your blood sugar measured?	1<br>2	No<br>Yes
815	815-1. Have you ever been told by a doctor or other health worker that you have raised blood sugar or diabetes?	1<br>2<br>77	No<br>**Yes** → 815-2<br>Don't know
	⇒ 815-2. In Yes, have you been told in the past 12 months?	1<br>2	No<br>Yes

Part9: Health information, Health seeking behavior (3 questions)

This is the last sections. We are going to ask about how to obtain the health information.

Health information, Health seeking behavior

901. Where do usually obtain the health information?(Mark all that apply)

1<br>Clinic/Hospital	2<br>CHW	3<br>Media(TV, Radio, IT)	4<br>Church	5<br>Friends	6<br>Family	7<br>Others	99<br>Don't know

902. When you are sick or you have some health problems, who do you talk to?(Mark all that apply)

1<br>Clinic/Hospital	2<br>CHW	3<br>Media(TV, Radio, IT)	4<br>Church	5<br>Friends	6<br>Family	7<br>Others	8<br>No one

903.	903-1. When you are sick, what do you do?	1<br>2<br>3<br>4<br>5<br>6<br>7<br>8	Go to clinic/hospital<br>See community health worker<br>Buy medicine at the *pharmacy*<br>Buy medicine at the *shop/market*(not pharmacy)<br>Take herbal medicine<br>See traditional healer<br>Do nothing<br>Others(Please specify) ( )
	⇒ 903-2. If answer ***except 1(not go to clinic)***, what is the reason you do not go to clinic? (Mark all that apply)	1<br>2<br>3<br>4<br>5<br>6<br>7<br>8<br>9	Far from home<br>Not enough money<br>No time because of work<br>No time (except work)<br>Long waiting time<br>Scared to go to clinic<br>Not trust health workers<br>Not trust western medicine<br>Others (Please specify) ( )

This is the end of the questions. Your cooperation will contribute very much to strengthen the health policy and improve people's health in Zambia.
Please say something if you have concern about health or any requests to health professional?

( )

Thank you very much for your cooperation!

S1 Appendix (Questionnaire: English)

Participant Feedback Form

Dear Participant,

We thank you very much for participating in our study of Risk Factors for Chronic Diseases, conducted by Kyoto University, Japan and University of Zambia. We would like to provide you with an overview of your results from the physical measurements.

____.______. 2016

Result

Blood Pressure	_____/_____ mmHg	□ Normal<br>□ Elevated<br>□ Raised<br>□ Currently on medication	SBP< 140 and DBP< 90<br>SBP 140-159 and/or DBP 90-99<br>SBP≧160 and/or DBP≧100
Height	_________ cm		
Weight	_________ kg		
Body Mass Index	_________ kg/m^2	□ Underweight<br>□ Normal weight<br>□ Overweight<br>□ Obese	BMI< 18.5<br>BMI 18.5-24.9<br>BMI 25-29.9<br>BMI≧30
Waist circumference	_________ cm		
Hip circumference	_________ cm		
Fasting blood sugar	_________ mg/dl	□ Normal<br>□ Raised<br>□ Currently on medication	< 126 mg/dl<br>≧126mg/dl
HbA1c	_________ %		
Total cholesterol	_________ mg/dl	□ Normal<br>□ Elevated<br>□ High	<190 mg/dl<br>190-239 mg/dl<br>≧240 mg/dl
HDL cholesterol	_________ mg/dl	□ Normal<br>□ Low	≧40 mg/dl (Men),≧50 mg/dl (Women)<br>< 40 mg/dl (Men), < 50 mg/dl (Women)
Triglycerides	_________ mg/dl	□ Normal<br>□ Raised	≧180mg/dl
Urinary sugar	(－) (±) (＋) (2＋) (3＋)		

This information is not a diagnosis for any conditions. It is for screening purposes only.
In case of any concerns, please consult a medical doctor.
