# Supplementary material for: Obesity matters but is not perceived: A cross-sectional study on cardiovascular disease risk factors among a population-based probability sample in rural Zambia
Source: PLoS One. 2018 Nov 29;13(11):e0208176. doi: 10.1371/journal.pone.0208176 (PMC6264511; doi:10.1371/journal.pone.0208176)
Supplement: S2 Appendix — (PDF) [file pone.0208176.s002.pdf]

S2 Appendix (Questionnaire: Local languages - Bemba, Nyanja, Tonga)

Socio-epidemiological study on risk factors of non-communicable diseases among adult population in rural Zambia

LOCATION AND DATE		
A.	Cluster number	└─┴─┴─┘
B.	Household number	└─┴─┴─┘
C.	Name and code of Village/compound	....
D.	Residential Classification	1: High Density<br>2: Low Density
E.	Language for interview	1: English<br>2: Bemba<br>3: Nyanja

VISIT RECORD				
	**1**	**2**	**3**	**Final visit**
**Interview Date**	(Day/Month)	(Day/Month)	(Day/Month)	(Day/Month)
**Physical/Biomedical Measurement Date**	(Day/Month)	(Day/Month)	(Day/Month)	(Day/Month)
**Interviewer's Name**				
**Result***				
***Result Codes**	1. Completed<br>2. Postponed<br>3. Refused<br>4. Household absent<br>5. Participants absent<br>6. Other(specify)			

CRITERIA			
001	How long have you lived in this village/town?		_________ months/years<br>Less than 6 months →not eligible
002	**For women:**<br>Are you pregnant?<br>Do you have a baby less than 6 months?	Yes<br>No	1<br>2
003	Is your age ***between 25 and 64 years old?***	Yes<br>No	1<br>2

Please circle the eligibility of this study

Eligible / Not Eligible

Physical Measurements

Blood Pressure

※*Make sure that participants have already seated calmly for* ***at least 15 minutes***

During the past two weeks, have you been treated for raised blood pressure with drugs (medication) prescribed by a doctor or other health worker?	Yes<br>No	1<br>2

Cuff size used

1: Small
2: Medium
3: Large

(mmHg)	Systolic	Diastolic
Reading 1	└─┴─┴─┘	└─┴─┴─┘
Reading 2	└─┴─┴─┘	└─┴─┴─┘
Reading 3	└─┴─┴─┘	└─┴─┴─┘

Height, Weight, Waist and Hip Circumference

Height	cm	└─┴─┴─┘.└─┘	Waist circumference	cm	└─┴─┴─┘.└─┘
Weight	kg	└─┴─┴─┘.└─┘	Hip circumference	cm	└─┴─┴─┘.└─┘

Biochemical Measurements

Since 8 pm yesterday**,** have you had ***anything to eat or drink***, other than water?	Yes<br>No	1<br>2
Today, have you **taken insulin or medications** prescribed by a doctor for ***raised blood glucose***?	Yes<br>No	1<br>2
During the past two weeks, have you been **taken medications** prescribed by a doctor for ***raised cholesterol***?	Yes<br>No	1<br>2

Blood test (Glucose/ Lipids)

Time of day blood specimen taken (24 hour clock)			Hours : minutes		└─┴─┘:└─┴─┘
Fasting blood glucose	mg/dl	└─┴─┴─┘.└─┘	Triglycerides	mg/dl	└─┴─┴─┘.└─┘
HbA1c	%	└─┴─┘.└─┘	LDL-Cholesterol	mg/dl	└─┴─┴─┘.└─┘
Total cholesterol	mg/dl	└─┴─┴─┘.└─┘	HDL Cholesterol	mg/dl	└─┴─┴─┘.└─┘

Urine test (Urinary Na/K ratio and urine sugar)

Time of day blood specimen taken (24 hour clock)		Hours : minutes	└─┴─┘:└─┴─┘
Urinary Na/K ratio	└─┴─┘.└─┘	Urinary sugar	1: (−)<br>2: (±)<br>3: (+)<br>4: (2+)<br>5: (3+)

S2 Appendix (Questionnaire: Local languages - Bemba, Nyanja, Tonga)

Part1: About yourself (14 questions)

We are going to ask about yourself and your family.

101.	What is your Gender?<br>Bushe muli baume nangula abanakashi?<br>Kodi ndinu amuna kapena akazi?<br>Hena muli basankwa naa bakaintu?	1<br>2	Male<br>Female
102.	How old are you?<br>Bushe muli nemyaka inga iyaku fyalwa?<br>Muli ndi dzaka zingati zakubadwa?<br>Mulaa myaka yongaye yakuzyalwa?		( ) years old
103.	Which ethnic groups do you belong to?<br>Bushe muli batundu shi?<br>Khondi dimwe atundu bwanji?<br>Muli bamushobo nzi?	1<br>2<br>3<br>4<br>5	Nyanja/Chewa/Ngoni/Tumbka/Nsenga<br>Bemba<br>Tonga<br>Lozi<br>Others (Please specify) ( )
104.	What is your marital status?<br>Bushe mwaba mucupo nangula awe?<br>Kodi muli mu banja?<br>Hena mulikwete naa mulikwetwe?	1<br>2<br>3<br>4<br>5<br>6	Never Married<br>Married<br>Married Living Separately<br>Divorced<br>Widow/Widowed<br>Never Married but Living Together
105.	How many people are living in your household?<br>Bushe mwikala banga mung'anda yenu?<br>Kodi pano pakhomo mukhala angati?<br>Mukala nyongaye mumukwashi wanu uuno?		( ) People
106.	How many people older than 15 years including yourself, live in your household?<br>Bushe nibanga ababa nemyaka 15 ukuya kumulu mung'anda yenu ukubikilako naimwe?<br>Kodi pakhomo pano muli angati omwe ali ndi zaka 15 kapena kuchilapo?<br>Bali bongaye mbomu kala limwi mumu kwashi uuno basika kumyaka 15 akwiindilila, kubikilizya andunwe mbomukala limwi?		( ) People
107.	In your household, who is handling the family budget?<br>Bushe munganda yenu ninani ulolekesha pami bofeshe yalupiya?<br>Pa banja lino kodi ndani ayanganira pa kasebenzesedwe ka ndalama munyumba mwanu?<br>Ngwani weendelezya makani amali mumukwashi wenu?	1<br>2<br>3	Myself<br>Husband/Wife<br>Other Family Member
108.	What is the highest level of education you have completed so far?<br>Bushe mwa pelela mu grade shani?<br>Muna pela pati kuma phunzilo?<br>Hena mwakasika mu bbuku nzi kwiiya cikolo?	1<br>2<br>3<br>4<br>5	No school at all<br>Basic school<br>Secondary school<br>College<br>More than College
109.	Which of the following best describes your main work status over the past 6 months?<br>Bushe ni chito shi mwabombapo pa myenshi 6 iyapita?<br>Pa myezi 6 yapita mwakala mukugwila nchito bwanji?<br>Kumyezi ili 6 yayinda eeyi, kapati kapati, mulimo nzi ngumwali kubeleka?	1<br>2<br>3<br>4<br>5<br>6<br>7	Government employed<br>Non-government employed<br>Self-employed (Self-business)<br>Student<br>Housewife/Househusband<br>Retired<br>Unemployed
110.	How much is your household income per month?<br>Bushe nishinga impiya mukwata pamwenshi mung'anda yenu?<br>Kodi mulandila ndalama zingati pakhomo pano pa mwezi?<br>Mujana mali manji buti amwezi mung'anda yanu?	1<br>2<br>3<br>4<br>5<br>6	Less than 500 Kwacha<br>500~999 Kwacha<br>1,000~1,999 Kwacha<br>2,000~2,999 Kwacha<br>More than 3,000 Kwacha<br>Don't know
111.	111-1. Are you taking any medication?<br>Bushe mulanwako umuti uli onse?<br>Kodi mukumwa mankhwala ali onse?<br>Hena kuli musamu ngomu silikwa?	1<br>2	No<br>**Yes**
	⇒ 111-2. If Yes, what kind of medication?<br>(Please specify your medication)<br>Nga mulesumina muti shi? (landeni umuti)<br>Ngati nitele, mukumwa mankhwala bwanji?<br>Kuti inzya, musamu zyi ngomubelesha?		→ **(** **)**<br>or 1. Hypertensive drug<br>2. Diabetic drug<br>3. ART (HIV treatment)<br>4. TB drug<br>5. Pain killer

112.	112-1. Are you suffering from any diseases?<br>Bushe mwalikwata ubulwele ubulibonse?<br>Kodi mudwala matenda ali onse?<br>Hena kuli bulwazi bumwi mbomu ciswa?	1<br>2	No<br>**Yes**
	⇒ 112-2. If Yes, what kind of diseases?<br>Nga mule sumina bu lwele shi?<br>Ngati nitelo, nimatenda bwanji?<br>Kuti inzya, mbulwazi nzi?		→ ( )
**For Women Only**			
113.	How many times were you Pregnant (Gravida) and Delivered (Parity)?<br>Bushe Mwakwatapo ama fumo imiku inga nokufyala imiku inga?<br>Kodi mu umoyo wanu mwakhala ndipathupi kangati, ndi kubeleka ana angati?<br>Hena mwamita akuzyala kwa zyiindi zyongaye?		Pregnant (Gravida)( )<br>Delivery (Parity) ( )
114.	114-1. Are you using any family planning method?<br>Bushe mulabofyako umuti uli onse uwa kukanya ukufyala?<br>Kodi musewenzesa njira ili yonse yolesa kubeleka?<br>Hena kuli zyila imwi njomubelesha yakukasha kumite?	1<br>2	No<br>**Yes**
	⇒ 114-2. If Yes, What kind of family planning method are you using?<br>Nga mule sumina, ninshila nshi mubonfya?<br>Ngati ndi thele, musewenzesa njira bwanji?<br>Kuti inzya, ninzila iili buti?		→ ( )

Part2: Food Security (9 questions)

We are going to ask about food issues in your household.

201.	201-1. In the past 30 days, did you worry that your household would not have enough food?<br>Bushe mushiku 30 ishapita, mwali sakamanapo ukweba ati munganda takuli ichakulya icha kumanina?<br>Pama siku 30 yapita, kodi munadodoma kuti banja lanu izakhala ilibe cakudya cokwanila?<br>Mumazuba 30 ayinda, hena kuli nimwaka tongooka kuti kunyina cakulya cikwene mumukwashi wenu?	1<br>2	No<br>Yes →
	⇒ 201-2. If Yes, how often did this happen in the past 30 days?<br>Nga mulesumina, bushe miku inga mushiku 30 iyapita ilyo chachi tikepo?<br>Ngati nditele, cinacitika kangati pama siku 30 yapita?<br>Na mbombubo, eeci cakacitika mubunji buti mazuba 30 ainda?	1<br>2<br>3	Rarely (1-2 times)<br>Sometimes (3-10 times)<br>Often (more than 10 times)
202.	202-1. In the past 30 days, were you or any household member not able to eat the kinds of foods you preferred because of a lack of resources?<br>Bushe mushiku 30 ishapita, mwali sangwapo ati imwe nangu umo mung'anda talilepo ifyakulya alefwaya pamulandu wakubulilwa ulupiya?<br>Pama siku 30 yapita, kodi imwe olo wabanja ali yense wamene ana kangiwa kudya cakudya comwe anafuna cifukwa cosowa ndalama?<br>Mumazuba 30 ayinda, hena nwebo antela umwi wamukwashi waka kakilwa kulya cakulya ciyandika nkaambo kakubula mali?	1<br>2	No<br>Yes →
	⇒ 202-2. If Yes, how often did this happen in the past 30 days?<br>Nga mulesumina, bushe miku inga mushiku 30 iyapita ilyo chachi tikepo?<br>Ngati nditele, cinacitika kangati pama siku 30 yapita?<br>Na mbombubo, eeci cakacitika mubunji buti mazuba 30 ainda?	1<br>2<br>3	Rarely (1-2 times)<br>Sometimes (3-10 times)<br>Often (more than 10 times)
203.	203-1. In the past 30 days, did you or any household member have to eat a limited variety of foods due to a lack of resources?<br>Bushe mushiku 30 ishapita, imwe olo uli onse munganda ali lyapo ifyakulya ifinono umulandu waku bulilwa?<br>Pama siku 30 yapita, kodi imwe olo wabanja ali yense wamene anadya zakudya zocepekela cifukwa chakusobedwa?<br>Mumazuba 30 ayinda, hena nwebo antela umwi wamukwashi wakalya cakulya citakweni nkambo kakubula mali?	1<br>2	No<br>Yes →
	⇒ 203-2. If Yes, how often did this happen in the past 30 days?<br>Nga mulesumina, bushe miku inga mushiku 30 iyapita ilyo chachi tikepo?<br>Ngati nditele, cinacitika kangati pama siku 30 yapita?<br>Na mbombubo, eeci cakacitika mubunji buti mazuba 30 ainda?	1<br>2<br>3	Rarely (1-2 times)<br>Sometimes (3-10 times)<br>Often (more than 10 times)
204.	204-1. In the past 30 days, did you or any household member have to eat some foods that you really did not want to eat because of a lack of resources to obtain other types of food?<br>Bushe mushiku 30 ishapita, imwe olo uli onse mung'anda alile ifyakulya ifyo tatemwene paumulandu waku bulilwa ulupiya?	1<br>2	No<br>Yes →

	Pama siku 30 yapita, kodi imwe olo ali yense wa banja kuli amene anadya cakudya comwe sanali kufuna cifukwa cosowa ndalama kuti mupeze zakudya zina?<br>Mumazuba 30 ayinda, hena nwebo antela umwi wamukwashi wakalya cakulya nchata yandi nkambo kakubula mali akuula cakulya cimwi?		
	⇒ 204-2. If Yes, how often did this happen in the past 30 days?<br>Nga mulesumina, bushe miku inga mushiku 30 iyapita ilyo chachi tikepo?<br>Ngati nditele, cinacitika kangati pama siku 30 yapita?<br>Na mbombubo, eeci cakacitika mubunji buti mazuba 30 ainda?	1<br>2<br>3	Rarely (1-2 times)<br>Sometimes (3-10 times)<br>Often (more than 10 times)
205.	205-1. In the past 30 days, did you or any household member have to eat a smaller meal than you felt you needed because there was not enough food?<br>Bushe mushiku 30 ishapita, kwalibapo ubushiku ilyo umo uwalupwa alilepo, aka kulya aka nono pamulandu wakubulilwa kwa fyakulya?<br>Pama siku 30 yapita, kodi pali imwe olo ali yense wa banja amene anadya mocepekela cifukwa sikunali cakudya cokwanila?<br>Mumazuba 30 ayinda, hena nwebo antela umwi wamukwashi wakalya cakulya cishoonto nkaambo kwakanyina cakulya cikwene?	1<br>2	No<br>Yes
	⇒ 205-2. If Yes, how often did this happen in the past 30 days?<br>Nga mulesumina, bushe miku inga mushiku 30 iyapita ilyo chachi tikepo?<br>Ngati nditele, cinacitika kangati pama siku 30 yapita?<br>Na mbombubo, eeci cakacitika mubunji buti mazuba 30 ainda?	1<br>2<br>3	Rarely (1-2 times)<br>Sometimes (3-10 times)<br>Often (more than 10 times)
206.	206-1. In the past 30 days, did you or any household member have to eat fewer meals in a day because there was not enough food?<br>Bushe mushiku 30 ishapita, kwalibapo ilyo imwe olo uwalupwa munganda alilepo ifyakulya imiku iyi nono mubushiku umulandu waku bulilwa kwa fyakulya?<br>Pama siku 30 yapita, kodi pali imwe olo ali yense wa banja amene anadya zakudya zing'ono cifukwa cocepekela kwa cakudya?<br>Mumazuba 30 ayinda, hena nwebo antela umwi wamukwashi wakalya cakulya kwa zyiindi zyishoonto nkaambo kwakanyina cakulya cikwnene?	1<br>2	No<br>Yes
	⇒ 206-2. If Yes, how often did this happen in the past 30 days?<br>Nga mulesumina, bushe miku inga mushiku 30 iyapita ilyo chachi tikepo?<br>Ngati nditele, cinacitika kangati pama siku 30 yapita?<br>Na mbombubo, eeci cakacitika mubunji buti mazuba 30 ainda?	1<br>2<br>3	Rarely (1-2 times)<br>Sometimes (3-10 times)<br>Often (more than 10 times)
207.	207-1. In the past 30 days, was there ever no food to eat of any kind in your house because of lack of resources to get food?<br>Bushe mushiku 30 ishapita, kwalipo ilyo tamwakwetepo ichakulya chili chonse munganda umulandu tamwa kwete impiya ukweba ati mushite ifya kulya?<br>Pama siku 30 yapita, kodi pali nthawi pomwe munalibe cakudya cili conse cifukwa cosowa chochita kuti musakile zakudya?<br>Mumazuba 30 ayinda, hena kuli nikwakanyina cakulya cili coonse mung'anda nkaambo kakubula mali akuula cakulya?	1<br>2	No<br>Yes
	⇒ 207-2. If Yes, how often did this happen in the past 30 days?<br>Nga mulesumina, bushe miku inga mushiku 30 iyapita ilyo chachi tikepo?<br>Ngati nditele, cinacitika kangati pama siku 30 yapita?<br>Na mbombubo, eeci cakacitika mubunji buti mazuba 30 ainda?	1<br>2<br>3	Rarely (1-2 times)<br>Sometimes (3-10 times)<br>Often (more than 10 times)
208.	208-1. In the past 30 days, did you or any household member go to sleep at night hungry because there was not enough food?<br>Bushe mushiku 30 ishapita, kwalibapo munganda uwalelepo ubushiku nensala umulandu tamwakwete ifya kulya ifya kumanina?<br>Pama siku 30 yapita, kodi pali imwe olo wabanja anagona njala cifukwa kunalibe cakudya cokwana?<br>Mumazuba 30 ayinda, hena nwebo antela umwi wamukwashi wakoona nzala nkaambo kwanyina cakulya cikwene?	1<br>2	No<br>Yes
	⇒ 208-2. If Yes, how often did this happen in the past 30 days?<br>Nga mulesumina, bushe miku inga mushiku 30 iyapita ilyo chachi tikepo?<br>Ngati nditele, cinacitika kangati pama siku 30 yapita?<br>Na mbombubo, eeci cakacitika mubunji buti mazuba 30 ainda?	1<br>2<br>3	Rarely (1-2 times)<br>Sometimes (3-10 times)<br>Often (more than 10 times)
209.	209-1. In the past 30 days, did you or any household member go a whole day and night without eating anything because there was not enough food?<br>Bushe mushiku 30 ishapita, kwalibapo munganda ukwi kalapo ukwabula ukulyapo ichakulya akasuba konse nobushiku umulandu waku bulilwa kwafya kulya.<br>Pama siku 30 yapita, kodi pali imwe olo wabanja anakhalapo siku lonse ndi usiku kopanda kudya cifukwa kunalibe cakudya cokwanila?<br>Mumazuba 30 ayinda, hena nwebo antela umwi wamukwashi waka kala buzuba boonse ama siku oonse kakunyina kulya cilicoonse nkambo kwakanyina cakulya cikwene?	1<br>2	No<br>Yes
	⇒ 209-2. If Yes, how often did this happen in the past 30 days?<br>Nga mulesumina, bushe miku inga mushiku 30 iyapita ilyo chachi tikepo?<br>Ngati nditele, cinacitika kangati pama siku 30 yapita?	1<br>2<br>3	Rarely (1-2 times)<br>Sometimes (3-10 times)<br>Often (more than 10 times)

	Na mbombubo, eeci cakacitika mubunji buti mazuba 30 ainda?		

Part3: Lifestyle (9 questions)

We are going to ask about your lifestyle and dietary habits.

Tobacco Use			
301.	301-1. Have you ever smoked tobacco?<br>Bushe mwalipepapo fyaka<br>Kodi munakokapo fodya<br>Hena kuli nimwaka fyeba tombwe?	1<br>**2**	No ⇒ Skip to 302<br>**Yes**
	⇒ 301-2. If yes, how often do you smoke tobacco?<br>Ngamulesumina, bushe miku inga mupepa?<br>Ngati mubvomekela, ndikambili bwanji?<br>Na mbombubo, mufweba kanji buti?	1<br>2<br>3<br>4	I have tried only a few times before<br>I'm a previous/Ex-smoker<br>I am a current smoker, but not every day<br>**I am currently a daily smoker**
	⇒ 301-3. If answer is 4, how many tobaccos do you smoke per day?<br>Nga mulesumina 4, bushe fwaka inga mupepa mubushiku?<br>Ngati mwayanka 4, mubema fodya ingati pasiku?<br>Na mbombubo, mufweba tombwe unji buti abuzuba?		**( )**
**Alcohol Use**			
302.	302-1. Have you ever drunk any alcohol?<br>Bushe mwali nwapo ubwalwa?<br>Kodi muna mwapo kale moba?<br>Hena kuli nimwaka nwide bukoko?	1<br>**2**	No ⇒ Skip to 303<br>**Yes**
	⇒ 302-2. If yes, how often do you drink alcohol?<br>Ngamulesumina, bushe miku inga munwa?<br>Ngati nditelo, ndi kambili bwanji komwe mukumwa moba?<br>Na mbombubo, munwa bukoko kwazyiindi zyinji buti?	1<br>2<br>3<br>4<br>5<br>6<br>7	Only a few times before<br>Once a month<br>2-3 times a month<br>1-2 days per week<br>3-4 days per week<br>Nearly everyday<br>Everyday
	⇒ 302-3. For what reasons do you drink alcohol? (Mark all that apply)<br>Bushi chinshi munwina ubwalwa?<br>Kodi ndicifukwa cani comwe cilengesa kuti inu mumwe moba?<br>Nkombonzi nchomu nwida bukoko?	1<br>2<br>3<br>4<br>5<br>6	To forget your problems<br>To relax<br>For enjoyment<br>To drink with friends/family<br>To help digestion<br>Others (Please specify) ( )

303~305. What do you think of smoking tobacco and taking alcohol?

	**303. Can affect you and your own health?**<br>Bushe kuti yaleta amafya ku bumi bwenu?<br>Kodi zingalete mabvuto ku umoyo wanu?<br>Hena inga caleta buyumu yumu kubuumi bwenu?		
	1<br>No	2<br>Yes	77<br>Don't know
**Tobacco**			
**Smoking around you**			
**Alcohol**			

	**304. Is it *good* for your health?**<br>Bushe cisuma kubumi bwenu?<br>Kodi ndicabwino ku umoyo wanu?<br>Hena nchibotu ku buumi bwenu?		
	**1**<br>**No**	2<br>Yes	77<br>Don't know
**Tobacco**			
**Smoking around you**			
**Alcohol**			

	**305. If *No*, how does it affect you and your own health?**<br>Nga mulekana, mafyanshi fingaleta ku bumi bwenu?<br>Ngati mukana, Kodi ndimabvuto otani zingalete ku umoyo wanu?<br>Naa mula kaka, mbuyumu nzi ku buumi bwenu?												
	1<br>Harm your lung	2<br>Harm your liver	3<br>Harm your stomach	4<br>Harm your heart	5<br>Harm your brain	6<br>Harm your kidney	7<br>Harm your vein	8<br>get TB	9<br>get cough	10<br>get family problem	11<br>get poverty	12<br>Others	13<br>Don't know
**Tobacco**													
**Smoking around you**													
**Alcohol**													

**Physical activity**			
306	306-1. Does ***your work*** involve intensity activity that causes ***increases in breathing or heart rate (like carrying or lifting heavy/light loads, digging or construction work, farming, etc.)*** for ***at least 10 minutes*** continuously? *(except commuting time)*<br>Bushe inchito yenu ila butusha umutima?<br>(Ukupemashika no kufuma umutima sana imiku iyingi muli 10 minutes)<br>Kodi nchito yomwe mugwira imaku thamangitsani mutima?<br>(kupema maningi naku tamanga mutima maningi munthawi ili 10 minites mokonkaniza)<br>Hena mulimo wenu ujatikizya kubelesha mubili kapati (Kamuyoya moyo kwindilizya kwaciindi 10 mamineti)?	1<br>**2**	No ⇒ Skip to 307<br>**Yes**
	⇒ 306-2. If Yes, how is the activity like?<br>Nga mule sumina, ni nchito iya shani?<br>Ngati muvomela, nchito ima nkala yotani?<br>Naa mula zumina, mulimo uli buti?	1<br>2	Vigorous (large workload)<br>Moderate (small workload)
	⇒ 306-3. What kind of activities do you do?<br>Bushe mubomba inchito shi?<br>Kodi nichito bwanji yomwe mugwila?<br>Mulimo nzi ngomucita?		( )
	⇒ 306-4. How many days in a typical week?<br>Bushe nishiku shinga mumulungu?<br>Masiku angati pamulungu?<br>Kwa zyiindi zyongayei mu mvwiki?		( )Days
	⇒ 306-5. How much time in a typical day?<br>Bushe nishita inga mubushiku?<br>Ma ola angati pa siku?<br>Kwazyiindi zyongaye buzuba bomwe?		( )hours
307	307-1. Do you do any intensity ***sports, fitness or recreational (leisure) activities*** that cause increases ***in breathing or heart rate (like running or football, cycling, swimming, netball, etc.)*** for at least 10 minutes continuously?<br>Bushe mulachita ama angalo aya kosa?<br>(Ukupemashika no kufuma umutima sana imiku iyingi muli 10 minutes)<br>Kodi mumacita za ma sewelera kapena zolimbisa thupi zamene ziku chosani thukuta?<br>(kupema maningi naku tamanga mutima maningi mutawo ili 10 minites mokonkaniza)<br>Hena mulacita zyi sobano zyiyumu kubelesha mubili?<br>(Kamuyoya moyo kwindilizya kwaciindi 10 mamineti)?	1<br>**2**	No ⇒ Skip to 308<br>**Yes**
	⇒ 307-2. If Yes, what kind of activities do you do?<br>Nga mule sumina, mucita miyangalo nshi?<br>Kodi nima sowela bwanji omwe aya?<br>Hena zyi sobano nzi eezi?		( )
	⇒ 307-3. How many days in a typical week?<br>Bushe nishiku shinga mumulungu?<br>Masiku angati pamulungu?<br>Kwa zyiindi zyongaye mu mvwiki?		( )Days
	⇒ 307-4. How much time in a typical day?<br>Bushe nishita inga mubushiku?<br>Ma ola angati pa siku?<br>Kwazyiindi zyongaye buzuba bomwe?		( )hours

308	308-1. Do you ***walk or use a bicycle*** for ***more than 10 minutes*** continuously to go to work or go somewhere in your daily life?<br>Bushe mwenda namolu nangula mubomfya injinga mukashita aka 10 minutes lyonse ukuya munchende nokufuma munchende?<br>Kodi mumayenda ndi myendo kapena kucova njinga kwa mphindi zili 10 popita kuli konse napo bwela?<br>Hena muleenda ama ulu olo naa anchinga kwa ciindi cili n10 kwiinka muzyi baka akuzya muzyi baka?<br>***This questions is focused on transport-related physical activity to travel around getting from place-to-place***	1<br>**2**	No ⇒ Skip to 309<br>**Yes**
	⇒ 308-2. If Yes, how many days in a typical week?<br>Bushe nishiku shinga mumulungu?<br>Masiku angati pamulungu?<br>Kwa zyiindi zyongaye mu mvwiki?		**(** **)**Days
	⇒ 308-3. How much time in a typical day?<br>Bushe nishita inga mubushiku?<br>Ma ola angati pa siku?<br>Kwazyiindi zyongaye buzuba bomwe?		**(** **)**hours
309	How many hours do you usually spend ***sitting or reclining*** <u>*except regular sleeping time*</u> in a typical day?<br>Bushe mubushiku nishita inga mwekalafye ukwabula ukubomba nangula ukulala fye?<br>Kodi ndi ma ola angati omwe mukhala olo kugona cabe posa cita cili conse pa siku?<br>Mutotola ciindi cilamfu buti kukala buyo antela kulyookezya abuzuba?<br>***Consider total time spent at work sitting, in an office, reading, watching television, using a computer, doing hand craft like knitting, resting etc.***		**(** **)**hours

Part4: Dietary habit (16 questions)

We are going to ask about your dietary habits in your daily meal.

Vegetables and Fruits

※ *In this section, we are asking about* ***green and colored vegetables and fruits*** *<u>(Not potatoes, grains or rice.)</u>*

401.	How many days do you eat <u>**fruits**</u> and <u>**vegetables**</u> in a typical week?<br>Bushe mulya ama fruits nama veji ishiku shinga mumulungu?<br>Kodi ndi masiku angati pa mulungu umodzi pomwe mukudya ma fruits ndi veji?<br>Hena mazuba ongaye muvwiki nomulya ma fruits ama veji?		Fruits: **(** **)** days<br>Vegetables: **(** **)** days

402. 402-1. Do you have any challenges to eat <u>**fruits**</u> and <u>**vegetables**</u>?
Bushe mwalikwata ubwafya ukulya ama fruits na ma veji?
Kodi muli ndi bvuto ili yonse kudya ma fruits ndi ma veji?
Hena mulaa penzi ili yoonse kulya ma fruits ama veji?

	1<br>No	2<br>Yes	77<br>Don’t know
**Fruits**			
**Vegetables**			

⇒ 402-2. If Yes, what are the main reasons of your challenges to eat <u>**fruits**</u> and <u>**vegetables**</u>? (Mark all that apply)
Nga mulesumina, bushe bwafya nshi icho tamulila ama fruits na ma veji?
Ngati nditele, ndi bvuto bwanji yomwe mulinayo kudya ma fruits ndi ma veji?
Muna ndi penzi nzi cipa kuti mubule kulya ma fruits ama veji?

	1<br>Don’t have money	2<br>Don’t like it	3<br>Don’t need it	4<br>Worry chemical use	5<br>Others (Specify)
**Fruits**					
**Vegetables**					

403. How important is it to you to eat <u>**fruits**</u> and <u>**vegetables**</u> every day?
Bushe chikankalashi kuli imwe ukulya ama fruits na veji mubushiku?
Kodi ndi ubwino bwanji omwe mupezamo mukudya ma fruits ndi veji tsiku ndi tsiku?
Hena mbubotu nzi bujanika mukulya ma fruits ama veji buzuba abuzuba?

	1<br>Not important at all	2<br>Not so important	3<br>Moderately important	4<br>Important	5<br>Very important
**Fruits**					
**Vegetables**					

Salad oil and fat

404.	404-1. Do you use oil or fat for your food/ cooking?<br>Bushe mubofya amafuta nangula salad mufyakulya mwipika?<br>Kodi muma sewenzesa mafuta olo salad paku phika chakudya chanu?<br>Hena mulabelesha saladi antela mafuta muli cakulya antela mukujika?	1<br>2<br>3	No ⇒ skip to 405<br>**Yes**<br>Don't know
	⇒ 404-2. If Yes, What type of fat is most often used for meal preparation in your household? (Mark all that apply)<br>Nga mulesumina, bushe mafutashi mubofya ilingi ukwipika mung'anda mumwenu?<br>Ngati nditele, nima futa bwanji omwe musewe nzesa po phika pano pa banja lanu?<br>Na mbombubo, mafuta ali buti kanji kanji ngomu belesha muku jika cakulya mumukwashi wenu? (Mark all that apply)	**1**<br>**2**<br>3<br>4<br>77	**Salad Oil**<br>**Butter or Margarine** (Including Blueband (brand name))<br>Animal Fat (Lard or Suet)<br>Others (Please specify) ( )<br>Don't Know
	⇒ 404-3. If you answer ***Salad Oil***, how much amount do you or your household use per day on average?<br>***(measure the amount of vegetable oil)***<br>Nga mwasumina kuli iyipusho 1, bushe mubomfya salad iyingi shani mung'anda mumwenu mubushiku bumo ilingi lingi?<br>Ngati mwayanka 1 (mafuta yama salad), niyambili bwanji mafuta yama veji imwe olo abanja amene musebenzesa pa tsiku kambili kambili?<br>Na mubelesha saladi, ngunji buti ngomubesha abuzuba nomujika kanji kanji?		How many days would it take to finish?<br>☐***750ml*** bottle of salad oil: ( )<br>☐***2.5L*** bottle of salad oil: ( )<br>☐***5L*** bottle of salad oil: ( )<br><br>If you don't you the bottle of salad oil, tell us specific amount per day.<br>☐ ( ) ml<br>☐ ( ) amounts of price
	⇒ 404-4. If you answer ***Butter or Margarine***, how much amount do you or your household use per day on average? ***(measure the weight of butter and margarine)***<br>Nga mulesumina munamba , bushe mubofya butter na margarine iyingi shani mung'anda yenu mubushiku?<br>Ngati mwayankha (mafuta ya butter olo margarine), niyambili bwanji mafuta ya butter olo margarine imwe olo abanja amene musebenzesa pa siku?<br>Na mubelesha bata na majarini,mubelesha munji buti abuzuba?		How many days would it take to finish?<br>☐***250g*** of butter and margarine: ( )

Salt

405.	How often is salt or salty seasoning added in ***cooking or preparing foods*** in your household?<br>Bushe nikanga mubikila limo salt olo salty seasoning kuchakulya chenu ilyo mulepekanya ukwipika?<br>Kodi nikangati kamene muyika salt olo salty seasoning popika olo pokonzekela chakudya munyumba yanu?<br>Nkanji buti nomulunga munyo nomujiaka mumukwashi wenu?	1<br>2<br>3<br>4<br>5<br>77	Never<br>Rarely<br>Sometimes<br>Often<br>Always<br>Don't know
406.	How often do you ***add salt or salty sauce*** on your food (dish) ***before or while you are eating***?<br>Bushe kengi shani ilyo mubika salt olo salty sauce kuchakulya chenu ilyo tamulalya olo pakulya?<br>Kodi nikangati kamene mumayika salt olo salty sauce kuzakudya zanu mukalibe kudya olo pakudya?<br>Henan kanji buti nomu bika munyo na soti muli cakulya kamu taninga lya antela nomulya?	1<br>2<br>3<br>4<br>5<br>77	Never<br>Rarely<br>Sometimes<br>Often<br>Always<br>Don't know
407.	On average, how much salt does your household consume per day?<br>Muku linganya, bushe mubomfya salt iyingi shani pabu shiku mung'anda mu mwenu?<br>Pantawi zambili, kodi niyambili bwanji munyu omwe muma sewenzesa mu banja lanu pa siku?<br>Kanji kanji, mukwashi wenu ulya munyo munji buti abuzuba?		measure the weight of salt _________ g
408.	408-1. Are you concerned of your salt intake?<br>Bushe mula posako amano kumilile ya salt?<br>Kodi ndinu okuzidwa nama dyedwe ya salt?<br>Hena kulya munyo cilamupa muzeezo?	1<br>**2**<br>3	No ⇒ skip to 409<br>**Yes**<br>Don't know
	⇒ 408-2. If yes, do you control your salt intake?<br>Nga mulesumina, bushe muleshako uku kontolola salt iyo mulya?<br>Ngati nditele, kodi muma chefyako kadyedwe ka salt?<br>Na mbombubo, hena kuli ntaamo zyomubweza kusola kucesha kulya kwa munyo?	1<br>**2**<br>3	No ⇒ skip to 409<br>**Yes**<br>Don't know
	⇒ 408-3. If yes, do you do any of the following on a regular basis to control your salt intake?<br>(Mark all that apply)<br>Nga mulesumina, bushe mula chitako ifyakonkapo ukukontolola imilile ya salt	1<br>2<br>3<br>4	Reduce the amount of salt intake<br>Reduce processed foods<br>Check the salt content of food labels<br>Buy low salt/sodium foods

	mucha kulya chenu?<br>Ngati nditele, kodi mumachita zosatiraazi kuti mu kontolole salt yamene mumadya?<br>Na mbombubo, hena kwa zyeezyi, kuli nchomucita kusola kucesha kulya munyo?	5<br>6<br><br>7	Use spices other than salt when cooking<br>Avoid eating foods prepared outside of home<br>Others (Please specify) ( )
**Sugar**			
409.	How often do you add sugar to your food or tea?<br>Bushe mubika sugar kanga kucha kulya chenu olo tea?<br>Kodi nikangati kamene mumayika sugar kuza kudya zanu olo tea?<br>Zyinji buti zyiindi nomubika sugar ku cakulya antela tea?	1<br>2<br>3<br>4<br>5<br>77	Never<br>Rarely<br>Sometimes<br>Often<br>Always<br>Don't know
410.	How much sugar do you consume per day?<br>Bushe ingi shani sugar mubonfya pabushiku?<br>Niyambili bwanji sugar yomwe musewe nzesa pa siku?<br>Hena ninji nbuti sugar njomubelesha abuzuba?		measure the weight of sugar ________ g

411~416. What do you think of your *salad oil, salt and sugar intake*?

	411. Do you think Zambians take a lot of these?<br>Bushe mumona kwati abena Zambia balabomfya sana ifi?<br>Kodi muona monga anthu a mu Zambia akudya kwambiri zinthu izi?<br>Hena muyeeya kuti bana Zambia bala belesha kapati eezyi?		
	1<br>No	2<br>Yes	77<br>Don't know
**Salad oil**			
**Salt**			
**Sugar**			

	412. How much of these do you think you consume?<br>Mukutontokonkanya kwenu, imilile ya ifi yaba shani?<br>Mukuganiza kwanu, ndizambiri bwanji mwa izi zomwe mumadya?<br>Hena muyeeya kuti mulya zyingi buti eezyi?					
	1<br>Too Much	2<br>A bit too much	3<br>Just the right amount	4<br>A bit too Little	5<br>Too Little	6<br>Don't know
**Salad oil**						
**Salt**						
**Sugar**						

	413. What is the main reason you take these? (Mark all that apply)<br>Bushe ninshi sana mulile fi?<br>Nicifukwa ninji maka maka mukudya izi?<br>Hena nkaambo nzi kanji kanji mulya zyeezyi?					
	1<br>Customs	2<br>Traditions	3<br>Taste	4<br>Health	5<br>Energy	6<br>Others (specify)
**Salad oil**						
**Salt**						
**Sugar**						

	414. Can affect you and your own health?<br>Bushe kuti fyamiletela amafya ku bumi?<br>Kodi zingakuleteleni bvuto pa umoyo wanu?<br>Hena zyileta penzi ku buumi bwenu?		
	1<br>No	2<br>Yes	77<br>Don't know
**Salad oil**			
**Salt**			
**Sugar**			

	415. Is it good your health?<br>Bushe cisuma ku buumi bwenu?<br>Kodi ndicabwino ku umoyo wanu?<br>Hena nchibotu ku buumi bwernu?		
	1<br>No	2<br>Yes	77<br>Don't know
**Salad oil**			
**Salt**			
**Sugar**			

	**416. If No, how does it affect you and your own health?**<br>Nga mulekana, mafyanshi fingaleta ku buumi bwenu?<br>Ngati mukana, Kodi ndimabvuto otani zingalete ku umoyo wanu?<br>Naa mulakazya, hena ndi penzi nnzi ku buumi bwenu?												
	1<br>Harm your liver	2<br>Harm your stomach	3<br>Harm your heart	4<br>Harm your brain	5<br>Harm your kidney	6<br>Harm your vein	7<br>get BP	8<br>get diabetes (sugar)	9<br>get over weight	10<br>get stroke	11<br>get heart disease	12<br>Others	13<br>Don't know
**Salad oil**													
**Salt**													
**Sugar**													

Part5: Food Safety (2 questions)

We are going to ask about what you think of the foods in the superstore or market.

501.

501-1. Do you think dietary habits have changed from the past (when you were young)?
Bushe mulemona kwati imilile nayi chinja uku chila akale?
Kodi muganiza kuti kadyedwe kachinja kuchila munthawi yakudala?
Hena muyeeya kuti kulya kwa cincha kwiinda mbukwa bede kaindi?

1 No
2 Yes → 501-2
3 Don't know

⇒ 501-2. If yes, what kinds of things have mostly changed ***in the market***? (Mark all that apply)
Nga mulesumina, bushe fintu shi mule mona kwati nafi chinja mu market?
Ngati ndi telo, kodi ndi chani chamene chachinja mu maliketi?
Na mbombubo, ninzi zyachincha kapati ku market?

1 Variety of food
2 Price
3 Increase fast-food
4 Increase processed food
5 Increase imported food
6 Others (Please specify) (   )

⇒ 501-3. What kinds of foods have ***increased*** and ***decreased*** from the past (when you were young?) (Mark all that apply)
Bushe fyakulya shi ifi fulile nefi chepele mumilile ukufuma mu kale (Ilyo mwali abaiche)?
Kodi nizakudya bwanji zamene zapaka ndi kuchepa kuchila muntawi yakudala (Pamene munali bafana)?
Nchakulya cili buti cacincha muku vula antela kucheya kuzwa ciindi cakaindi (kuzya nimwa cili bana bashoonto)?

※ *This question* ***is not*** *asking of your availability of food, asking* ***the food selling in the market.***

	1<br>Salt	2<br>Sugar	3<br>Salad oil	4<br>Animal fat	5<br>Fish	6<br>Vegetable	7<br>Fruits	8<br>Potatoes	9<br>Beans
**Increased**									
**decreased**									

	10<br>Groundnuts	11<br>Bread	12<br>Snacks	13<br>Juice	14<br>Fast-food	15<br>Processed-food	16<br>Imported food	17<br>Others(specify)
**Increased**								
**decreased**								

502.

502-1. Do you have any concern about foods that are sold in the market?
Bushe mula sakanamapo pafyakulya ba   shitishiwa mu market?
Kodi ndi mwe okhuzidwa pa zakudya zomwe zigulisidwa ku maliketi?
Hena mujisi butongosi kujatikizya cakulya cuulisigwa mu market?

1 No
2 Yes → 502-2
3 Don't know

⇒ 502-2. If yes, ***What kind of food*** are you mostly ***concern***? (Mark all that apply)
Nga mulesumina, bushe fyakulya nshi pali ifi ifimisakamika ilingi lingi?
Ngati ndi tele, ndi zakudya bwanji zomwe ziku khuzani maka maka?
Naa mbombubo, kanji kanji, nchicili buti cakulya cimupa matongoosi?

1<br>Vegetable	2<br>Fruits	3<br>Chicken	4<br>Beef	5<br>Fish	6<br>Other meats	7<br>Restaurant	8<br>Fast-food	9<br>Selling outside	10<br>Others (specify)

⇒ 502-3. What are ***your concerns about food***? (Mark all that apply)
Bushe finshi ifimisakamika pafyakulya?
Kodi ndi zichani zokukhuzani pa zakudya?
Matongoosi nzi ngomu jisi azyakulya?

1 Chemical
2 Injection (to animals)
3 Fertilizer
4 Food quality
5 Food poison
6 Others (Please specify)   (   )

S2 Appendix (Questionnaire: Local languages - Bemba, Nyanja, Tonga)

Part6: Mental Stress and relationship of neighborhood (12 questions)

We are going to ask about the psychological distress and stress management.

Psychological stress

※These questions concern how you have been feeling ***over the past 30 days.***

No.	Question	None of the time	A little of the time	Some of the time	Most of the time	All of the time
601.	How often did you feel nervous?<br>Bushe nikanga ilyo mumfwa umwenso?<br>Ndi kambili bwanji pomwe mumanvela manta?<br>Mbunji buti bwazyiindi nimwa limvwa kuyoowa?	1	2	3	4	5
602.	How often did you feel hopeless?<br>Bushe nikanga ilyo mumfwa ati tamukwete ichichetekelo?<br>Ndi kambili bwanji pomwe mumanvela kopanda ciye mbekedzo?<br>Mbunji buti bwazyiindi nimwa limvwa kubula lushomo?	1	2	3	4	5
603.	How often did you feel restless or fidgety?<br>Bushe nikanga ilyo mufya kwati teti mutushe olo uku kana kwi kali kana?<br>Ndi kambili bwanji pomwe mumanvela osapumula olo osa khazikika?<br>Mbunji buti bwazyiindi nimwa limvwa kubula kulyookezya?	1	2	3	4	5
604.	How often did you feel depressed that nothing could cheer you up?<br>Bushe nikanga ilyo mumfya uku pwililila sana icha kweba ati tapali necha kumumfwisha bwino?<br>Ndi kambili bwanji mumazinvela okhumudwa ndi kuphanda comu kondwelesani?<br>Mbunji buti bwazyiindi nimwa limvwa kukatala kwa mizezo kakunyima cimupa kukondwa?	1	2	3	4	5
605.	How often did you feel that everything was an effort?<br>Bushe nikanga ilyo mumfya ati fyonse kubikako amano ukweba ati fichitike?<br>Ndi kambili bwanji pomwe mumazi nvela kuti zonse ndi kucita kuyesa kuti vicitike?<br>Mbunji buti bwazyiindi nimwa limvwa kuti zyoonse zyakusola?	1	2	3	4	5
606.	How often did you feel worthless?<br>Bushe nikanga ilyo mufya ati tamwakwata inchito?<br>Ndi kambili bwanji pomwe mumanzinvela osakhala ndi phindu ili yonse?<br>Mbunji buti bwazyiindi nimwa limvwa kubula mpindu?	1	2	3	4	5

Stress management

607.

607-1. Do you feel any stress in your daily life?
Bushe mulofya uku chepelwa mubumi bwenu?
Kodi munvela kukhumudwa mu umoyo wanu?
Hena mulimvwa kukatala mumizeezo mubuumi bwenu?

1	No ⇒ skip to 708
**2**	**Yes**
3	Don't know

⇒ 607-2. If Yes, what is currently the main cause of stress in your life? (Mark all that apply)
Nga mulesumina, bushe nishi mufyila uku chepekelwa mubumi bwenu ilingi lingi?
Ngati ndi tele, kodi cacikulu ndi cani cipasa kukhumudwa mu umoyo wanu?
Naa mbombubo, nchinzi cimupa kukala mumi zeezo mubuumi bwenu kanji kanji?

1	Family
2	Relationships
3	School
4	Work
5	Money
6	Health

⇒ 607-3.What do you do to reduce your stress? (Mark all that apply)
Bushe fishi muchita uku pwisha kunfwa uku chepekelwa?
Ndi ciyani comwe muma chita ku chefya kukhumudwa?
Hena mucita buti kusola kucesha kukatala kwa mu mizeezo?

1	2	3	4	5	6	7	8	9	10
Exercise	Talk to friends/family	Eat	Talk to Health workers	Drink some alcohol	Pray	Reading bible	Sleep	Don't do anything	Others (specify)

Social cohesion and trust

No.	Question	Strongly disagree	Disagree	Neither Agree nor Disagree	Agree	Strongly agree
608.	People around here are willing to help their neighbors<br>Abantu ebo mwikala nabena balai pelesha ukwa fyilisha ama neighbors<br>Anthu kuno ndi omasuka ku thandidza anzao<br>Bantu kuno bala lipa kugwashana amboba kala limwi	1	2	3	4	5

609.	This is a close-knit neighborhood<br>Abantu mumushi balikatana capamo<br>Uyu ndi munzi ogwiridzana<br>Kuli lukamantano kuno	Strongly disagree	Disagree	Neither Agree nor Disagree	Agree	Strongly agree
		1	2	3	4	5
610.	People in this neighborhood can be trusted<br>Abantu mu mushi muno baliba aba chishinka<br>Anthu kuno ndi okulupilika<br>Bantu kuno balashomeka	Strongly disagree	Disagree	Neither Agree nor Disagree	Agree	Strongly agree
		1	2	3	4	5
611.	People in this neighborhood generally don't get along with each other<br>Abantu mu mushi uno tabofwana<br>Anthu kuno samvelana<br>Bantu kuno taba mvwani	Strongly disagree	Disagree	Neither Agree nor Disagree	Agree	Strongly agree
		1	2	3	4	5
612.	People in this neighborhood do not share the same values<br>Abantu ababa mu mushi uno tabakwata imikalile yimo yene<br>Anthu kuno ali ndi ka khalidwe kosiyana<br>Bantu kuno taba jisi muzeezo omwe	Strongly disagree	Disagree	Neither Agree nor Disagree	Agree	Strongly agree
		1	2	3	4	5

Part7: About overweight/Obesity (8 questions)

We are going to ask about your perception of overweight/Obesity.

Obesity			
701.	What do you think of your body weight?<br>Ukulingana noku fina kwenu, bushe mulemona kwati mwaba pe?<br>Kulingana ndi momwe mu lemela pa sikelo, kodi muganiza muli pati?<br>Kujatikizya mubili wenu, hena mulimvwa kuti muli ali?	1<br>2<br>3<br>4	Underweight<br>Normal weight<br>Overweight<br>Very Overweight(Obese)
702.	Do you think Zambians prefer to be overweight?<br>Bushe mumona kwati ama Zambians bali temwa ukwina sana?<br>Kodi muganiza anthu amu Zambia akonda kulema thupi?<br>Hena muyeeya kuti bana Zambia bala yanda kuneneeya kapati?	1<br>2<br>77	No<br>Yes<br>Don't know
703.	Do you prefer to be overweight?<br>Bushe kuti mwatemwa ukwina sana?<br>Kodi munga konde kuina maningi?<br>Hena muyanda kuneneeya kapati?	1<br>**2**<br>77	No<br>**Yes** → 704-1<br>Don't know
704.	⇒ 704-1. If yes, what is the main reason why you prefer to be overweight? (Mark all that apply)<br>Nga mulesumina, bushe nishi icho mu-temenwa uku ina?<br>Ngati ndi tele, ndi cifukwa chanji mukonda kune nepa?<br>Na mbombubo, nchinzi ncomuyanda kuneneeya?	1<br>2<br>3<br>4<br>**5**<br>6<br>77	Attractive<br>Looks wealthy<br>Culture/tradition<br>Avoid to be seen poor<br>**Scared of being seen to be sick** → 704-2<br>Others (Please specify) ( )<br>Don't know
	⇒ 704-2.If answered 5, what kind of sickness are you scared of being seen?<br>Nga mwachi sumina 5, bushe ma lwele nshi mutinapo pali aya uku mimona ukulwala?<br>Ngati mwayanka 5, kodi ndi matenda bwanji omwe muyopa ku fanana nayo?<br>Naa muyoowa kuyeyelwa kuciswa 5, mbulwa zi nzi?	1<br>2<br>3<br>4<br>5	Tuberculosis<br>Malaria<br>HIV<br>High blood pressure<br>Others (Please specify) ( )

S2 Appendix (Questionnaire: Local languages - Bemba, Nyanja, Tonga)

705.	Please see the pictures below.<br>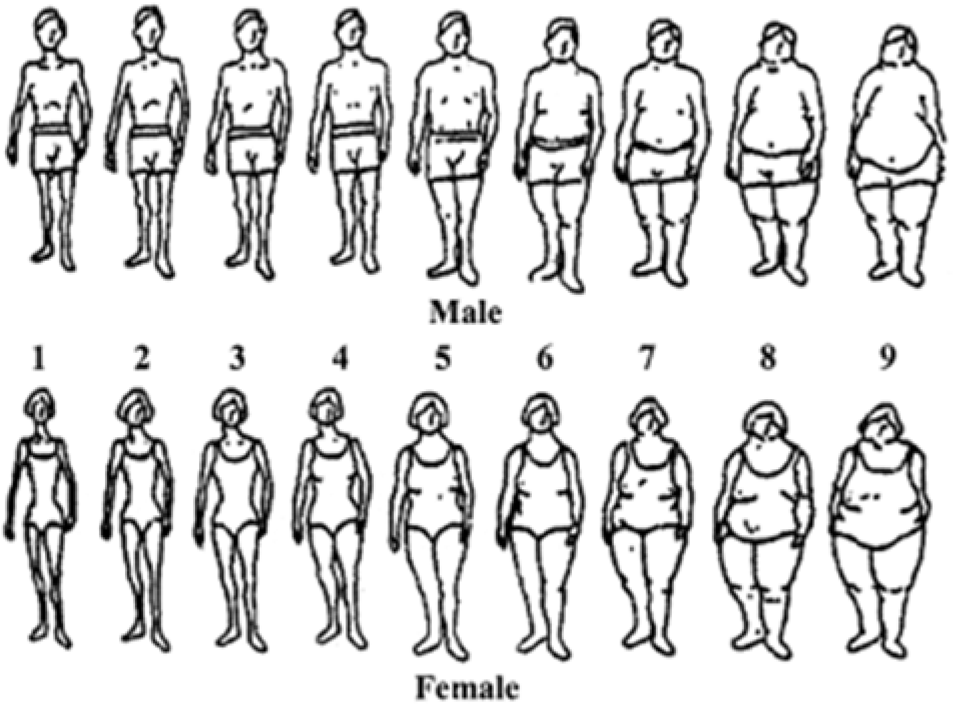 		
	705-1. Which number of figure is the closest of your body size?<br>Bushe ninamba shi iyo mwabapo umubili wenu?<br>Kodi ndi ci thunzi thunzi citi mufanana nacho?<br>Hena inga mwaleelanya acimbonimboni cili?	1 2 3 4 5 6 7 8 9	
	705-2. Which number of ***male*** figure do you recognize overweight?<br>Bushe ninamba nshi iya mwaume uyi nine sana?<br>Ndi ci thunzi thunzi citi ca mwamuna comwe ndi conenepa koposa?<br>Nchi fwanikiso cili camwaalumi cinenede kwiinda?	1 2 3 4 5 6 7 8 9	
	705-3. Which number of ***female*** figure do you recognize overweight?<br>Bushe ninamba shi iya mwanakashi uyi nine sana?<br>Ndi ci thunzi thunzi citi ca mukazi comwe ndi onenepa koposa?<br>Nchi fwanikiso cili camukaintu cinenede kwiinda?	1 2 3 4 5 6 7 8 9	
706.	How important is having a normal body weight to you?<br>Bushe cika nkala shani uku ikala na mubili waina bwino?<br>Kodi ndi cabwino bwanji kukhala ndi thupi yonenedpa bwino kwa inu?<br>Nchibotu buti kuneneeya kweelede kulindunwe?	1<br>2<br>3<br>4<br>5	Not important<br>Not so important<br>Moderately important<br>Important<br>Very important

707~708. What do you think of stress and obesity?

	707. Can these affect you and your own health?<br>Bushe kuti fyamiletela amafya ku bumi?<br>Kodi zingakuleteleni bvuto bwanji pa umoyo wanu?<br>Hena eezyi inga zyamu letela mapenzi nzi mu buumi bwenu?		
	1<br>No	2<br>Yes	77<br>Don’t know
**Stress**			
**Obesity**			

	708. If Yes, how does it affect you and your own health?<br>Nga mule sumina, kuti fya yafya shani imwe no buumi bwenu?<br>Ngati muvomela, kodi zinga kudze bwanji inu ndi umoyo wanu?<br>Naa mula zumina, inga zyaja tikizya buti ndunwe abuumi bwenu?												
	1<br>Harm your liver	2<br>Harm your stomach	3<br>Harm your heart	4<br>Harm your brain	5<br>Harm your kidney	6<br>Harm your vein	7<br>get BP	8<br>get diabetes (sugar)	9<br>get over weight	10<br>get stroke	11<br>get heart disease	12<br>Others	13<br>Don’t know

**Stress**													
**Obesity**													

Part8: Cardiovascular diseases (15 questions)

We are going to ask about your knowledge and perception of cardiovascular diseases.

801~811. Please answer the following question?

	801. Do you know the term of following diseases?<br>Bushe mwaliumfwako amalwele aya?<br>Kodi munamverako za matenda aya<br>Hena makamvwa zyama lwazi aaya?		
	1<br>No	2<br>Yes	77<br>Don't know
**High BP**			
**Stroke**			
**Heart disease**			
**Diabetes**			

⇒ **If No, or don't know at each disease, skip the questions of each disease.**

	802. Are you concerned about developing these diseases?<br>Bushe cila misakamikapo kuti mwakwata amalwele aya?<br>Kodi ndinu okhuzidwa kuti mungakhale ndi matenda aya?<br>Hena cilamu yoosha kuti inga mwa ciswa malazi aaya?		
	1<br>No	2<br>Yes	77<br>Don't know
**High BP**			
**Stroke**			
**Heart disease**			
**Diabetes**			

	803. Can these transmit between people?<br>Bushe kuti ya yambukira kubantu?<br>Kodi muganiza ayambukila pakati pa anthu?<br>Hena aaya malwazi alatambukila?		
	1<br>No	2<br>Yes	77<br>Don't know
**High BP**			
**Stroke**			
**Heart disease**			
**Diabetes**			

	804. Are these dangerous than infectious diseases like HIV?<br>Bushe mumona kwati ubulwele bwali bipapo ukuchila akashishi?<br>Kodi muganiza ndiyo yopsa kwambili kucila matenda ya kali yonde yonde?<br>Hena alayoosha kwiinda malwazi atambukila mbuli sikaleke?				
	1<br>Strongly disagree	2<br>Disagree	3<br>Neither Agree nor Disagree	4<br>Agree	5<br>Strongly agree
**High BP**					
**Stroke**					
**Heart disease**					
**Diabetes**					

	805. What increase the risk to develop these diseases?<br>Bushe finshi ifinga fusha umo ukukwata ama lwele aya?<br>Kodi ndicani cingaculukise kuti munthu atenge matenda aya?<br>Hena cinzi cinga cuvuzya muntu kuti abwezi malwazi aaya?										
	1<br>Smoking	2<br>Alcohol	3<br>Stress<br>(thinking a lot)	4<br>Being over-weight	5<br>Getting older	6<br>Too much salt	7<br>Too much oil	8<br>Too much sugar	9<br>Poor diet	10<br>physically inactive	11<br>High BP	77<br>Don't know
**High BP**												
**Stroke**												
**Heart disease**												
**Diabetes**												

S2 Appendix (Questionnaire: Local languages - Bemba, Nyanja, Tonga)

	806. Can these diseases be prevented?<br>Bushe amalwele aya kuti ya chingilishiwa?<br>Kodi mathenda aya yanga chingilidziwe?<br>Hena malwazi aaya inga akwa bililwa?		
	1<br>No	2<br>Yes	77<br>Don't know
**High BP**			
**Stroke**			
**Heart disease**			
**Diabetes**			

	807. <u>If Yes,</u> how can it be prevented?<br>Nga mulesumina, bushe kuti bwa chingilisha shani?<br>Ngati ndi thele, kodi yanga chingi lizidwe bwanji?<br>Hena inga yakwa biliwa buti?							
	1<br>Improving diet	2<br>Taking medications	3<br>Doing exercise	4<br>Losing weight	5<br>Quit smoking	6<br>Nothing	7<br>Others	77<br>Don't know
**High BP**								
**Stroke**								
**Heart disease**								
**Diabetes**								

	808. Have you ever learned before?<br>Bushe mwali sambililapo akale?<br>Kodi muna khalapo kale ndi punzilo pa mathenda aya?<br>Hena mwaka yiisigwa kale?		
	1<br>No	2<br>Yes	77<br>Don't know
**High BP**			
**Stroke**			
**Heart disease**			
**Diabetes**			

	809. <u>If Yes</u>, Where did you learn?<br>Nga mwalisa mbililapo, bushe nikwisa?<br>Ngati ndi telo muna punzilila kuti?<br>Na mbombubo, mwaka iya kuli?				
	1<br>Clinic/Hospital	2<br>CHW	3<br>Media(TV, Radio, IT)	4<br>Church	99<br>Others
**High BP**					
**Stroke**					
**Heart disease**					
**Diabetes**					

	810. Are these diseases common in Zambia?<br>Bushe mumona kwati ama lwele aya yengi mu Zambia?<br>Kodi muganiza kuti aya mathenda ndi ochuluka mu Zambia?<br>Hena aaya malwazi manji mu Zambia?		
	1<br>No	2<br>Yes	77<br>Don't know
**High BP**			
**Stroke**			
**Heart disease**			
**Diabetes**			

S2 Appendix (Questionnaire: Local languages - Bemba, Nyanja, Tonga)

	811. Is there anyone who has these diseases in your family?<br>Bushe eko ali mulupwa ukwete ama lwele aya?<br>Kodi kuli imodzi wabanja ali nayo matenda aya?<br>Hena nkwali wamu kwashi ujisi malwazi aaya?		
	1<br>No	2<br>Yes	77<br>Don't know
**High BP**			
**Stroke**			
**Heart disease**			
**Diabetes**			

History of Raised Blood Pressure and Diabetes

812.	812-1. Have you ever had your blood pressure measured?<br>Bushe mwali pimishapo BP?<br>Kodi muna pimisapo kale BP?<br>Hena kuli nimwaka pimidwe BP?	**1**<br>**2**	**No**<br>**Yes**
	⇒ 812-2. If yes, where did you measure your blood pressure last time?<br>Nga mule mule sumina, nikwisa mwi piminwe BP?<br>Ndati muvomela, ndi kuti komwe muna pimisa BP nthawi yatha?<br>Nkuli nkumwaka pimidwe BP ciindi camana?		
	⇒ 812-3. What is the main reason that made you check your blood pressure last time?<br>Bushe chinshi ichalengele ukweba ati bamipime BP?<br>Kodi ndi cifukwanji muna pimisa PB nthawi yatha?<br>Cinzi cakapa kuti mupimwe BPciindi camana?	1<br>2<br>3<br>4<br>5	Regular checkup of hypertension *(already diagnosed high BP)*<br>I had symptoms related to blood pressure<br>Checked blood pressure when I visited clinic for other diseases<br>Health checkup *(not regular checkup)*<br>Others (Please specify) ( )
	If No,<br>⇒ 812-4. What is the main reason that made you not to check your blood pressure before?<br>Chinshi calengelwe kuti mwipimwa BP?<br>Ndi cifukwa chanji mukalibe kupimisapo BP kumbuyo ko?<br>Ninzi cakapa kuti muta pimwi BPciindi camana?	1<br>2<br>3<br>4<br>5<br>6<br>7	Don't have time<br>Don't know where to access service<br>Don't think it is important<br>Don't know how<br>Don't have a chance<br>Feel scared to check<br>Others (Please specify) ( )
813.	813-1. Have you ever been told by a doctor or other health worker that you have raised blood pressure or hypertension?<br>Bushe bali mwebako kuli ba dokota olo bambi aba lolekesha pabumi ukutila namukwata BP?<br>Kodi kuli a dotolo ngakhale aku cipatala omwe ana muuzanipo kuti BP yanu yakwedza?<br>Hena kuli nimwaka ambilwa adokota kuti mujisi BP itantide?	1<br>2<br>77	No<br>**Yes**<br>Don't know
	⇒ 813-2. If Yes, have you been told in the past 12 months?<br>Nga mule sumina, bushi bali myebapo pameshi shapita 12?<br>Ngati nitele, kodi ana muzana pamyezi 12 yapita?<br>Naa mulazumina, baka mwaambila myezi 12 yayinda?	1<br>2	No<br>Yes
814	Have you ever had your blood sugar measured?<br>Bushe bali mipimapo bulwele bwa shugar?<br>Kodi ana mupimani matenda ya shugar?<br>Hena baka mupima bulwazi bwa shugar?	1<br>2	No<br>Yes
815	815-1. Have you ever been told by a doctor or other health worker that you have raised blood sugar or diabetes?<br>Bushe bali mwebapo ba docta nangula aba shonfi ba ku chipatala kuti mwali kwata shugar yenu nainina?<br>Kondi ana mu uzani a dotola kapena osewenza ku chipatala kuti shugar yanu yakwela?<br>Hena baka mwaambila ba dokota antle babeleka ku cibbadela kuti shugar yanu ya tanta?	1<br>2<br>77	No<br>**Yes**<br>Don't know
	⇒ 815-2. In Yes, have you been told in the past 12 months?<br>Nga mule sumina, bushi bali myebapo pameshi shapita 12?<br>Ngati nitele, kodi ana muzana pamyezi 12 yapita?<br>Naa mulazumina, baka mwaambila myezi 12 yayinda?	1<br>2	No<br>Yes

812-2 response options:

1<br>Clinic/Hospital	2<br>Workplace	3<br>School	4<br>Home	5<br>Friend's place	6<br>Others

S2 Appendix (Questionnaire: Local languages - Bemba, Nyanja, Tonga)

Part9: Health information, Health seeking behavior (3 questions)

This is the last sections. We are going to ask about how to obtain the health information.

Health information, Health seeking behavior

901. Where do you usually obtain the health information?(Mark all that apply)
Bushe nikwisa yeko musenda ama sambilila pali imikalile ya bumi?
Kodi ndikuti komwe muma thenga uthenga wa za umoyo?
Hena mujana kuli twaambo tujatikizya buumi?

1 Clinic/Hospital	2 CHW	3 Media(TV, Radio, IT)	4 Church	5 Friends	6 Family	7 Others	99 Don't know

902. When you are sick or you have some health problems, who do you talk to?(Mark all that apply)
Bushe nga mwalwala niba nani ebo mulashana nabena pa bwafya bwama lwele yenu?
Kodi ngati mwa dwala ndipo mulindi vutho ya umoyo, muma lankula ndi ndani?
Kuti kaabe mwaciswa antela tamulivwide kabotu mubuumi bwenu, ngwani ngo mwambaula anguwe?

1 Clinic/Hospital	2 CHW	3 Media(TV, Radio, IT)	4 Church	5 Friends	6 Family	7 Others	8 No one

903.

Question	Code	Answer
903-1. When you are sick, what do you do?<br>Bushe ilyo mulwala, fishi muchita?<br>Kodi ngati mwadwala, muma cita ciani?<br>Naa mwaciswa, mucita buti?	1<br>2<br>3<br>4<br>5<br>6<br>7<br>8	Go to clinic/hospital<br>See community health worker<br>Buy medicine at the *pharmacy*<br>Buy medicine at the *shop/market*(not pharmacy)<br>Take herbal medicine<br>See traditional healer<br>Do nothing<br>Others(Please specify) ( )
⇒ 903-2. If answer ***except 1(not go to clinic)***, what is the reason you do not go to clinic?<br>(Mark all that apply)<br>Nga mulesumina, yambi uku chila 1, nishi tamwila ku clinic?<br>Ngati mwayanka kuchila 1(simupita ku cipatala), ndi chifukwa chiyani simupita ku cipatala?<br>Naa mulazumina, kunze ya 1, Nkambo nzi tamuunki ku cibbadela?	1<br>2<br>3<br>4<br>5<br>6<br>7<br>8<br>9	Far from home<br>Not enough money<br>No time because of work<br>No time (except work)<br>Long waiting time<br>Scared to go to clinic<br>Not trust health workers<br>Not trust western medicine<br>Others (Please specify) ( )

This is the end of the questions. Your cooperation will contribute very much to strengthen the health policy and improve people's health in Zambia.
Please say something if you have concern about health or any requests to health professional?

( )

Thank you very much for your cooperation!
